# Deficit drip irrigation and nitrogen fertigation synergistically modulate sesame performance: morpho-physiological, agronomic, and quality traits

**DOI:** 10.1186/s12870-026-09548-w

**Published:** 2026-07-20

**Authors:** Asmaa Hamoda, Mokhtar Dabbour, Nora Hussein

**Affiliations:** 1 Department of Agronomy, Faculty of Agriculture, Benha University, P.O. Box 13736, Moshtohor, Qaluobia Egypt; 2 Department of Agricultural and Biosystems Engineering, Faculty of Agriculture, Benha University, P.O. Box 13736, Moshtohor, Qaluobia Egypt

**Keywords:** Deficit drip irrigation, Nitrogen fertigation, Sesame, Chlorophyll a, Relative water content, Oil content, Radar plots

## Abstract

The indiscriminate and excessive application of nitrogen fertilizer, coupled with over-irrigation, represents a well-documented driver of reduced crop productivity and severe environmental degradation in agroecosystems. Although deficit drip fertigation is recognized for enhancing water and nutrient use efficiency, quantitative evidence regarding its optimal irrigation and nitrogen fertigation levels for sesame remains critically lacking. Therefore, this study was conducted to quantitatively assess the effectiveness of deficit drip irrigation regimes (I_50_: 50, I_80_: 80, and I_100_: 100% of ET_c_) and nitrogen fertigation rates (N_25_: 25, N_75_: 75, and N_100_: 100% of the recommended nitrogen dose) on the morpho-physiological, agronomic, and quality attributes of sesame over two growing seasons of 2024 and 2025. ANOVA revealed that irrigation level, nitrogen fertigation rate, and their interaction had highly significant effects (*p* < 0.001) on leaf number, dry weight of capsules, chlorophyll b, capsule number, and seed yield. Full irrigation (100% ET_c_) substantially improved plant height, leaf number, and SPAD value of sesame. The maximum capsule dry weight and capsule number were observed under the 75% nitrogen treatment, suggesting that excessive nitrogen may promote vegetative growth at the expense of reproductive development. The application of N_100_ under well-watered conditions (I_100_) significantly enhanced chlorophyll a, chlorophyll b, and relative water content (1.98 mg·g⁻¹ FW, 0.827 mg·g⁻¹ FW, and 89.03%, respectively) compared to other treatments. Notably, the highest capsule number per plant (93.33) and 1000-seed weight (3.80 g) were documented under the interactive effects of full irrigation and a 75% nitrogen fertigation, which consequently maximized seed yield (1570.51 kg·ha⁻¹). Conversely, water stress (I_50_) considerably reduced nitrogen and oil content, especially at 25% nitrogen fertigation, indicating a decrease in the nutritional quality of sesame seeds. Furthermore, multivariate analyses substantiated that the application of N_75_ under full irrigation achieved an optimal balance among crop productivity and quality. These outcomes provide practical guidance for farmers seeking to maximize both sesame productivity and its potential profitability.

## Introduction

Sesame is extensively utilized across the food, pharmaceutical, and nutraceutical industries in many countries, primarily due to its high content of oil, protein, and bioactive antioxidants. The seeds are nutritionally dense, containing oil (36–63%), protein (18–28%), carbohydrates (14–16%), digestible fiber (9–11%), and minerals (5–7%) such as calcium and phosphorus [1, 2]. In developing regions, sesame serves as a valuable alternative cash crop for smallholder farmers, contributing substantially to livelihood improvement, nutritional security, and food preservation. Accordingly, global consumption of sesame seeds reached $7.40 billion in 2024, with oil accounting for 62.10% of this market value [3]. Despite its worldwide economic importance, sesame cultivation in Egypt is still limited, covering only 43,201 hectares with an average yield of 0.97 tons per hectare in 2024 [4], a value significantly lower than the global yields of 1.48–1.84 tons per hectare achievable under optimal agronomic management [5, 6]. This marked gap between its widespread international adoption and persistently low domestic output underscores the urgent need for implementing effective agronomic practices to expand both cultivated area and productivity.

However, realizing this economic potential is fundamentally constrained by water availability, the most limiting factor for crop growth in Egypt, despite the proximity of the Nile River. Notably, the agricultural sector accounts for 80–90% of freshwater use, most of which is directed toward crop production [5]. This challenge is exacerbated by climate change and growing water scarcity, particularly in semi-arid and arid regions [7]. Under arid summer conditions, climate change intensifies these effects through prolonged elevated temperatures and sustained water stress. Such synergistic stress disrupts morphological, biochemical, physiological, and molecular processes in plants, adversely affecting plant height and root biomass [8], photosynthetic efficiency [9], and yield parameters such as capsule number, seed number per capsule, and seed weight [10, 11]. Under water stress, an 11.78% yield reduction was observed in sesame [12], owing to impaired carbohydrate translocation and cell turgor, which accelerated floral abortion and reduced pollen viability and seed yield. Consequently, the growing threat of freshwater shortages and the increasing frequency of drought, driven by climate change, have motivated researchers to adopt water-saving strategies aimed at producing more crop per drop. In that sense, expanding sustainable irrigation techniques, such as drip irrigation (DI), substantially improves crop yields and addresses global food security concerns. The DI system has proven particularly effective at reducing energy consumption compared to sprinkler irrigation systems [13], while enhancing crop production and water use efficiency across various crops [14, 15]. Additionally, relative to traditional surface irrigation, DI decreases field water consumption by 30–50% by minimizing evaporation and seepage losses [16]. This system has therefore become a crucial tool for sustainable agriculture, especially in water-scarce areas. Bastug et al. [17] reported that the highest sesame oil content was achieved under the DI_100_ treatment (100% of crop water requirement), reaching 52.9% in 2014 and 57.7% in 2015, while the DI_75_ treatment yielded a similarly high value (57.6%) in 2015. Furthermore, DI system facilitates better fertilizer management and more efficient nutrient distribution directly to the root zone of plants through drippers, resulting in less plant stress and higher crop yields and quality [18]. Building on the efficiency of DI, the integration of fertigation, defined as the application of fertilizers through the irrigation system, further enhances resource use precision.

Fertigation is an efficient technique that delivers liquid or soluble fertilizers through irrigation systems, enabling dose optimization based on plant developmental stage and nutrient uptake [19], thereby reducing production costs and preventing pollution from over-fertilization. Drip fertigation maintains optimal moisture and nutrient conditions within the crop root zone [20], enhancing application uniformity and nutrient use efficiency, while minimizing nitrate leaching compared to conventional methods. In arid and semi-arid soils, the effectiveness of conventional fertilizers is often limited by nutrient fixation and reduced availability. Recent studies demonstrate that the use of advanced nutrient carriers and optimized fertilizer application strategies can significantly improve nutrient bioavailability, strengthen antioxidant defense systems, and enhance overall crop yield and productivity [21]. Nitrogen, a key component of proteins, chlorophyll, and hormones, promotes the accumulation of photosynthetic assimilates in plants [22]. Previous reports indicate that the growth and yield of sesame are greatly influenced by nitrogen application, with optimal seed yield typically achieved at 46–100 kg N·ha⁻¹ across major sesame-producing regions [23]. Blal et al. [24] reported that the maximal seed yield per plant (36.90 g) was recorded at 95 kg N·ha⁻¹. Moreover, Couch et al. [19] noted that 31–66% of absorbed nitrogen was remobilized to capsules and seeds during maturation. However, high nitrogen application can exacerbate water stress in sesame. Given that both irrigation and nitrogen fertilization are key determinants of sesame production, their optimization is essential.

Previous research on deficit drip irrigation and nitrogen fertigation has predominantly focused on the single-treatment effects on crop productivity in semi-arid and arid regions. However, the synergistic effects of deficit drip irrigation and nitrogen fertigation on the morpho-physiological responses, yield components, and seed quality of sesame have not been quantitatively characterized under Egyptian conditions. We hypothesized that (1) a moderate nitrogen reduction (75% of the recommended rate) under full irrigation would optimize the source-sink balance and maximize yield, and (2) deficit irrigation would increase crop water productivity but reduce seed quality. Therefore, the present study investigated the combined effects of irrigation levels (100, 80, and 50% of crop evapotranspiration) and nitrogen fertigation rates (25, 75, and 100% of the recommended dose) on the growth, physiological traits, yield-related attributes, chemical composition, and crop water productivity of sesame. The relationships between measured sesame characteristics and treatment variables were also examined using radar plots, principal component analysis, Pearson’s correlation, and hierarchical clustering analysis. The findings offer a practical and sustainable strategy for farmers to stabilize sesame productivity under water-scarce conditions.

## Materials and methods

### Experimental site and meteorological data

**Fig. 1:**
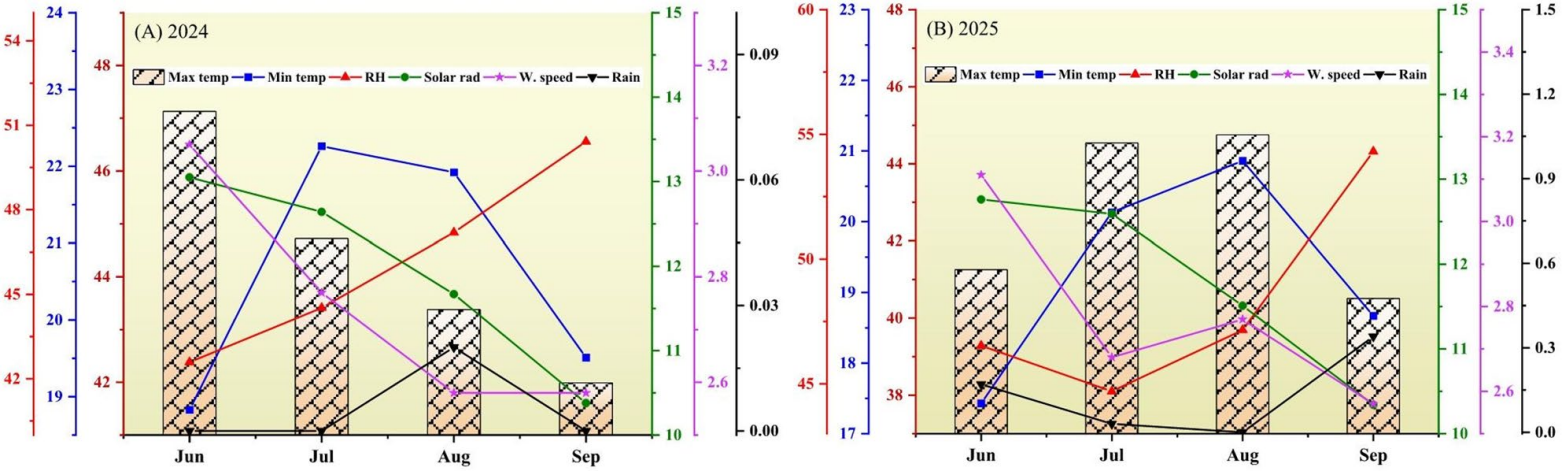
Monthly meteorological data for the study site during the 2024 (**A**) and 2025 (**B**) growing seasons: maximum and minimum temperature (°C), solar radiation (MJ·m⁻²·d⁻¹), relative humidity (%), wind speed (m·s⁻¹), and rainfall (mm). Data were obtained from the NASA POWER database (https://power.larc.nasa.gov/data-access-viewer/)

A two-year field study (2024–2025) was conducted at the Research and Experimental Station, Faculty of Agriculture, Benha University, Egypt (31.10° E longitude and 30.45° N latitude). Before the experiment in each of the two growing seasons, composite soil samples were collected using an auger from two depths (0–40 cm and 40–70 cm) at randomly selected locations within the experimental site to assess the chemical and hydro-physical properties of the soil. These depths were selected based on site-specific root distribution and soil constraints. In sesame, most fine roots and nutrient uptake occur within the upper 60 cm of the soil profile. Additionally, a compacted plow pan at ~ 65–70 cm functionally restricts root penetration, rendering depths beyond 70 cm largely root-free, with negligible contribution to sesame nutrition. The collected samples were air-dried, pulverized, and sieved (2-mm mesh), and three subsamples from each depth were subsequently taken for analysis. The chemical and hydro-physical properties were examined as described previously [25–28], and the results are presented in Table 1. Furthermore, the meteorological data during the growing seasons of sesame (June-September) are displayed in Fig. 1. The mean solar radiation (11.87 MJ·m⁻²·d⁻¹) and maximum temperature (43.54 °C) provided adequate photosynthetically active radiation to support both vegetative and reproductive development in sesame. The mean minimum temperature of 19.95 °C, though conducive to early root and shoot development, influenced phenological dynamics and initial growth rates. Moreover, the mean wind speed of 2.76 m·s⁻¹ enhanced canopy aeration and reduced leaf surface humidity, thus lowering disease pressure. The critically low precipitation (0.07 mm) recorded over the four-month growing season, coupled with a relative humidity of 43.53%, underscored the necessity of precise irrigation to meet the crop water requirement.

**Table 1:** Physicochemical and hydro-physical properties of the experimental site

Depth (cm)	Physicochemical properties											
	EC (dS·m⁻¹)						pH (1:2.5 w/v)					
0–40	0.568						7.723					
40–70	0.742						7.879					
	Soluble cations (mmol_c_·L⁻¹)						Soluble anions (mmol_c_·L⁻¹)					
	Ca^2+^	K^+^		Na^+^	Mg^2+^		Cl^-^		CO_3_²⁻	HCO_3_^-^		So_4_²⁻
0–40	2.102	0.800		3.223	2.404		1.497		0.000	2.601		4.416
40–70	1.500	1.348		3.895	1.301		2.303		0.000	1.900		3.854
	Particle size distribution (%)											
	Fine sand		Coarse sand			Silt		Clay			Soil texture	
0–40	20.947		1.280			27.922		49.851			Clay	
40–70	21.231		1.963			28.188		48.618			Clay	
	Hydro-physical properties											
	Field capacity (%)		Permanent wilting point (%)			Available water (%)		Hydraulic conductivity (cm·h⁻¹)			Bulk density (g·cm⁻^3^)	
0–40	33.504		16.001			17.496		1.190			1.104	
40–70	38.497		18.499			20.002		0.423			1.150	

Data presented for each depth represent the mean values from the two seasonal assessments

### Experimental field management

The field was prepared to a fine tilth through sequential operations, including disc harrowing for primary tillage, soil pulverization, and leveling. Prior to sowing, calcium superphosphate (15.5% P₂O₅) was broadcast at a rate of 476 kg·ha⁻¹. Sesame (*Sesamum indicum* L. cv. Shandawil 3) was used in this research, and the seeds were obtained from the Agricultural Research Center, Ministry of Agriculture and Land Reclamation, Egypt. This cultivar originated in Egypt in 1987 as a line selected from the cross Giza 32 × N. A. 130. It is an indeterminate, photoperiod-sensitive, high-yielding cultivar widely cultivated in arid and semi-arid regions. Furthermore, Shandawil 3 exhibits pronounced drought tolerance traits, as evidenced by a low stress susceptibility index (SSI = 0.55), a low tolerance index (TOL = 36.92), a high stress tolerance index (STI = 0.94), and a high relative drought index (RDI = 1.03) [29]. The seeds were manually sown at a rate of 5 kg·ha⁻¹ on June 3, 2024, and June 6, 2025, at a depth of 2 cm, and harvested in September of both seasons. Sowing was carried out in hills spaced at 60 cm inter-row spacing and 10 cm intra-row spacing. Emergence percentage, calculated as the number of emerged seedlings at 10 days after sowing (DAS) divided by the total number of seeds sown, averaged 71.3% across all plots. After thinning to two plants per hill at 14 days after emergence, the final stand count was recorded at 30 DAS by counting all established plants per plot, yielding a mean density of 299,700 plants·ha⁻¹ (equivalent to ~ 90% of the thinned plant population). All other agricultural practices followed regional standards for sesame cultivation.

### Experimental design and layout

**Fig. 2:**
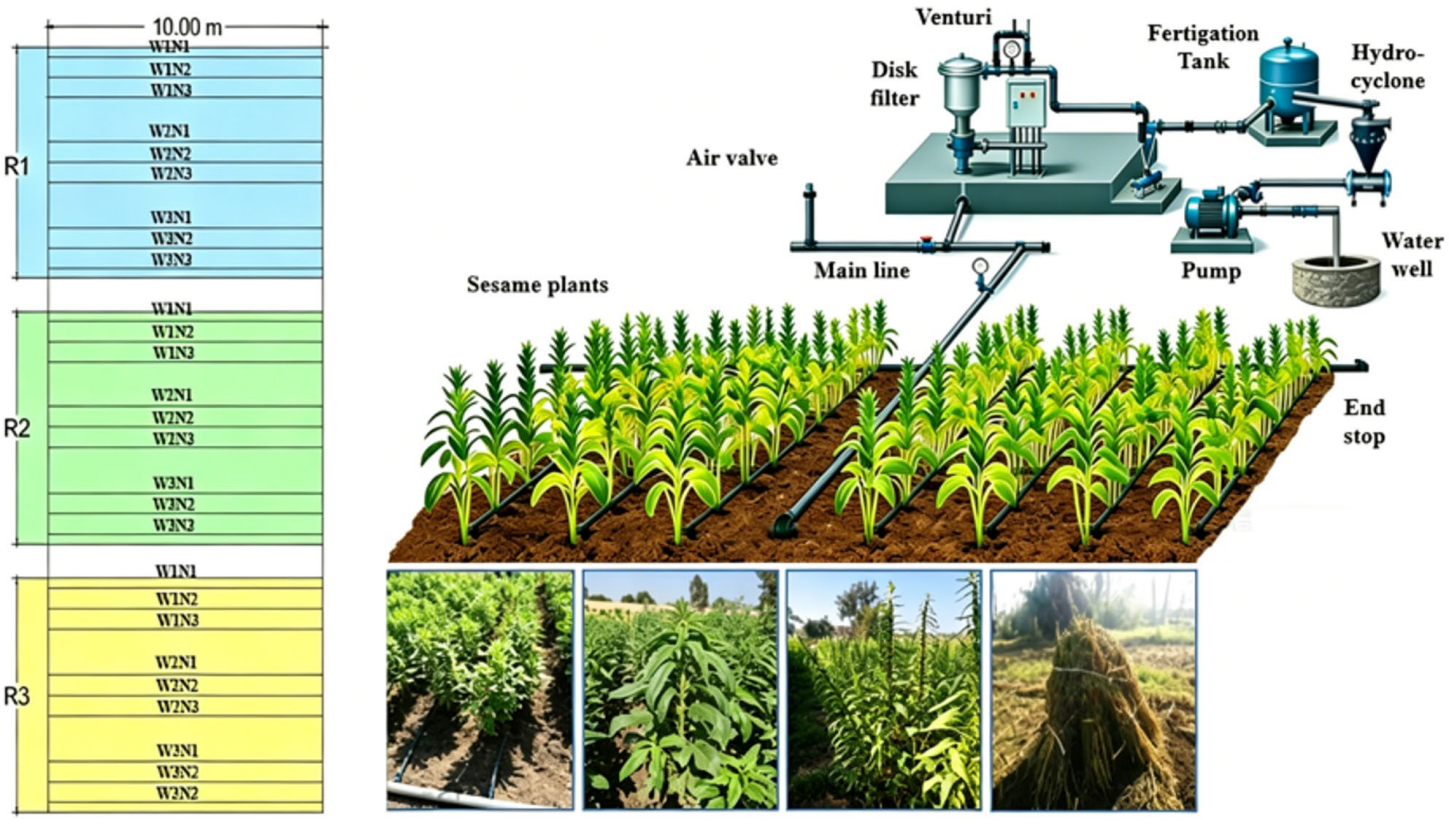
Schematic illustration of the experimental design used to assess the effects of deficit drip irrigation and nitrogen fertigation on sesame

A field experiment was conducted over two successive growing seasons to evaluate the agronomic response of sesame to different nitrogen application rates under deficit drip irrigation. The experimental layout was a split-plot arrangement in a randomized complete block design with three replications per season. Three irrigation levels (100, 80, and 50% of crop evapotranspiration (ET_c_)) were allocated randomly to the main plots. The 100% ET_c_ treatment represented full irrigation (well-watered condition), while the 80% and 50% treatments were applied to stimulate moderate and severe water deficits, respectively, thereby simulating water stress conditions. The seasonal irrigation water requirement for sesame at 100% ET_c_ was calculated as 6169.75 m³·ha⁻¹, as detailed in the subsequent irrigation water requirement section (Table 2). The sub-plots were assigned to nitrogen fertigation treatments at 25%, 75%, and 100% of the recommended nitrogen dose for sesame (95.0 kg N·ha⁻¹), according to the guidelines of the Ministry of Agriculture and Land Reclamation, Egypt. Nitrogen was supplied as ammonium nitrate (33.5% N) via a Venturi injector integrated into the drip irrigation system. Nitrogen fertigation was initiated at 21 DAS, corresponding to the mid-vegetative stage; the second split was applied at 35 DAS (early flowering stage), and the third split was applied at 49 DAS (capsule formation stage). The specific nitrogen rates applied were 23.8, 71.3, and 95.0 kg N·ha⁻¹ for the 25%, 75%, and 100% treatments, respectively. Ammonium nitrate was used in compliance with local safety regulations. In regions where its use is restricted, calcium ammonium nitrate (26–27% N) or urea (46% N) combined with appropriate nitrification inhibitors can serve as functionally equivalent alternatives to provide comparable nitrogen supply in drip-irrigated systems. The experiment consisted of three irrigation levels and three nitrogen rates, with three replications, resulting in a total of 27 experimental plots. Each plot consisted of four ridges, each 10 m long with an inter-ridge spacing of 60 cm, giving a plot area of 24 m². A schematic illustration of the experimental design used to assess the combined effects of drip irrigation and nitrogen fertigation on sesame is exhibited in Fig. 2.

### Irrigation water requirement

The amount of irrigation water applied to the sesame under drip irrigation was calculated according to the method of Vermeiren and Jobling [30], as follows:

$$\:IW=\frac{{ET}_{o}\times\:Kc\times\:\prod\:}{Ea\times\:(1-LR)}\times\:10$$

$$\:{ET}_{c}={ET}_{o}\times\:Kc$$

where IW is the irrigation water applied under drip irrigation system (m^3^·ha⁻¹·irrigation⁻¹); ET_o_ is the reference evapotranspiration, which was computed from onsite meteorological data (mm·day⁻¹); Kc is the crop coefficient, with values are as follows: initial stage = 0.31, developmental stage = 0.75, mid-season stage = 0.98, and late-season stage = 0.51 [31]; $\:\prod\:$ is the irrigation interval (day); and Ea is the drip irrigation efficiency (0.90); and LR is the leaching requirement (assumed as 10% of the total amount of water, m^3^·ha⁻¹·irrigation⁻¹). The **s**easonal ET_o_, Kc, ET_c_, and irrigation water applied (IW) for sesame across two growing seasons are presented in Table 2.

**Table 2:** Seasonal ET_o_, Kc, ET_c_, and irrigation water applied (IW) for sesame across two growing seasons

Season 1						Season 2					
Date	Growth stages	Et_o_	Kc	Et_c_	IW (mm)	Date	Growth stages	Et_o_	Kc	Et_c_	IW (mm)
Jun 3	Initial stage	7.3	0.31	2.26	19.56	Jun 6	Initial stage	7.7	0.31	2.39	20.63
Jun 10		7.3		2.26	19.56	Jun 13		8.1		2.51	21.70
Jun 17		7.2		2.23	19.29	Jun 20		7.9		2.45	21.16
Jun 24	Developmental stage	7.1	0.75	5.33	46.02	Jun 27	Developmental stage	7.5	0.75	5.63	48.61
Jul 1		7.1		5.33	46.02	Jul 4		7.2		5.40	46.67
Jul 8		7.2		5.40	46.67	Jul 11		7.4		5.55	47.96
Jul 15		7.1		5.33	46.02	Jul 18		7.3		5.48	47.31
Jul 22	Mid-season stage	7.3	0.98	7.15	61.82	Jul 25	Mid-season stage	7.5	0.98	7.35	63.52
Jul 29		6.6		6.47	55.90	Aug 1		7		6.86	59.28
Aug 5		6.4		6.27	54.20	Aug 8		6.7		6.57	56.74
Aug 12		6.5		6.37	55.05	Aug 15		6.8		6.66	57.59
Aug 19		6.5		6.37	55.05	Aug 22		6.7		6.57	56.74
Aug 26	Late-season stage	6.2	0.51	3.16	27.33	Aug 29	Late-season stage	6.6	0.51	3.37	29.09
Sep 2		5.7		2.91	25.12	Sep 5		6.1		3.11	26.89
Sep 9		5.8		2.96	25.56	Sep 12		6.1		3.11	26.89
Total IW = 603.17 mm (= 6031.7 m^3^·ha⁻¹)						Total IW = 630.78 mm (= 6307.8 m^3^·ha⁻¹)					

### Irrigation system and installation

The drip irrigation (DI) system and its installation were designed in accordance with standard engineering procedures and specifications. It comprised a 3 HP electrical centrifugal pump equipped with 50.8 mm diameter pipes for both suction and delivery. The pumping discharge was 24 m^3^·h⁻¹ at an operating pressure head of 27 m. A control gate valve, pressure gage, and flow meter were installed on the pump discharge line. A standard and reliable Venturi injector (1-inch diameter, threaded connections) was incorporated, operating within a pressure range of 1–4 bar and requiring a pressure differential of 0.5–1 bar for effective fertilizer suction, a critical design requirement for the irrigation system. PVC pipe lines (63 mm diameter) were used to convey and distribute irrigation water from the source to the supply laterals (drip lines). The laterals were made of 16 mm diameter polyethylene tubing, with drippers spaced at 30 cm intervals, each providing a discharge rate of 4 L·h⁻¹.

## Data collection and measurements

### Growth traits

At 75 DAS, five plants were randomly sampled from each sub-plot to assess growth parameters. Plant height was measured in centimeters from the soil surface to the apical meristem. The numbers of leaves and branches per plant were also counted. The stems, leaves, and capsules were oven-dried (60 °C, 72 h) until a constant weight was achieved, after which their mean dry weight was determined (in grams).

### Physiological attributes

#### Relative chlorophyll content (SPAD)

The relative chlorophyll content was measured using a SPAD-502 chlorophyll meter (Minolta, Osaka, Japan) on the third fully expanded leaf from the apex of the main stem (counting the first fully expanded leaf as position 1). All measurements were taken between 9:00 and 11:00 AM to minimize diurnal variation. Three plants per plot were randomly selected, and for each plant, three leaves at the specified position were assessed. On each leaf, three readings were taken and averaged to obtain a single SPAD value per leaf.

### Chlorophyll a and b content

The protocol of Arnon [32], with some modifications, was followed to determine chlorophyll a and b contents. Fresh leaf tissue (0.5 g) from each experimental plot was homogenized in 20 mL acetone (80%). The homogenate was filtered, and the absorbance of the supernatant was then read at 663 nm and 645 nm using a spectrophotometer. Chlorophyll a and b contents were computed as follows:

$$\:Chlorophyll\:a\:\left(mg.g^{-1}FW\right)=\left[\left(12.7\times\:{A}_{663}\right)-\left(2.69\times\:{A}_{645}\right)\right]\times\:\frac{{V}_{t}}{m\times\:1000}$$

$$\:Chlorophyll\:b\:\left(mg.g^{-1}FW\right)=\left[\left(22.9\times\:{A}_{645}\right)-\left(4.7\times\:{A}_{663}\right)\right]\times\:\frac{{V}_{t}}{m\times\:1000}$$

where V_t_ (mL) is the total extract volume; m (g) is the fresh weight of the sample; and A_663_ and A_645_ are the absorbance values at the respective wavelengths.

### Relative water content

The relative water content (RWC) of sesame plants was evaluated following the method described by González and González-Vilar [33]. Leaf discs were excised from the mid-laminal region of the leaves and weighed immediately (FW). The discs were thereafter immersed in distilled water for 4 h at room temperature, surface-dried with absorbent paper, and reweighed to obtain the turgid weight (TW). The samples were then oven-dried (70 °C,72 h) to determine the dry weight (DW). The RWC (%) was calculated as follows:

$$\:RWC\:\left(\%\right)=\frac{FW-DW}{TW-DW}\times\:100$$

### Yield and yield components

At physiological maturity (120 DAS), plants were manually harvested at the onset of capsule dehiscence. The plants were cut at the stem base and sun-dried until all capsules fully dehisced. Following drying, the number of capsules per plant was quantified by counting all capsules from the five plants, and the average was subsequently calculated. Capsules length was measured on five randomly selected capsules taken from the middle of the main stem. To determine the 1000-seed weight (g), five random samples of 1000-seed per plot were cleaned and weighed, and the mean value was recorded. For seed yield, all cleaned seeds per plot were weighed, and the resulting weight was converted to kg·ha⁻¹.

### Chemical analysis

The oil content of sesame seeds was determined based on the method of Hamoda and Dabbour [34] with slight modifications. The sample powder (5 g) was loaded into a cellulose thimble and subjected to Soxhlet extraction. A 150 mL of petroleum ether was added to a pre-weighed, dry round-bottom flask, which was then equipped with the extractor. The system was thermally controlled at 65 °C using a heating mantle to provide a condensation drip rate of 2–3 drops per second for 3 h. Preliminary experiments confirmed that extraction beyond three hours yielded less than 0.5% additional oil, validating that a three‑hour Soxhlet extraction was sufficient for near‑complete oil recovery. After the extraction, the solvent was evaporated, and the recovered sesame oil was oven-dried at 105 °C to remove any residual solvent. The flask was subsequently cooled to ambient temperature in a desiccator and reweighed. The oil content was determined as follows:

$$\:Oil\:content\:\left(\%\right)=\frac{{m}_{1}-{m}_{2}}{{m}_{1}}\times\:100$$

where m_1_ and m_2_ (g) represent the mass of the sample before and after extraction.

The nitrogen content of sesame seeds was examined using the Kjeldahl method [35]. Sample powder (0.20 g) was digested in a heat-resistant tube at ~ 380 °C with sulfuric acid and a catalyst mixture of potassium sulfate and copper (II) sulfate until a colorless solution was achieved. The mixture was cooled and diluted with distilled water to 100 mL. An aliquot of the resulting solution (15 mL) was distilled with 30 mL of NaOH (40%). The released ammonia was trapped in boric acid (4%) with methyl red and bromocresol green and then titrated with 0.1 N HCL. A blank was prepared using the same reagents without the sample. The nitrogen content (N; %) were computed as follows:

$$\:N\:\left(\%\right)=\frac{\left({V}_{1}-{V}_{2}\right)\:\times\:\:C\:\times\:\:0.014}{m\:\times\:\:({V}_{a}/{V}_{d})}\times\:100$$

where V_1_ and V_2_ (mL) denote the volume of HCL titrated for the sample and blank, respectively; C (mol·L⁻¹) denote the concentration of HCL; V_a_ denotes the aliquot volume taken for distillation (15 mL); and V_d_ denotes the total digest volume (100 mL); and m (g) denotes the weight of sesame sample.

### Crop water productivity

Crop water productivity (CWP) was calculated according to Michael [36] using the following equation:

$$\:CWP\:\:(kg.{m}^{-3})=\:\frac{Total\:sesame\:seed\:yield\:}{Total\:irrigation\:water\:applied}\:$$

### Statistical analysis

Results are expressed as means ± standard deviation from three independent experimental replications. Prior to performing a combined analysis of variance (ANOVA), the assumptions of normality and homogeneity of variances were evaluated using the Shapiro-Wilk test and Levene’s test, respectively, on the residuals of all response variables. These assumptions were verified separately for each year and were all satisfied (*p* > 0.05), rendering data transformation unnecessary. Subsequently, a combined ANOVA with years and treatments as fixed effects was conducted using MSTAT-V21 software, followed by Tukey’s post-hoc test (*p* < 0.05) to identify significant differences among treatment means. Multivariate analyses, including Pearson’s correlation, heatmaps, radar plots, and principal component analysis (PCA), were performed using OriginPro 2023b software (OriginLab Corporation, Northampton, MA, USA) [41, 42].

## Results and discussion

### Vegetative growth

ANOVA results (Table 3) revealed significant main effects of irrigation levels and nitrogen fertigation on most growth parameters of sesame, except for branch number. Irrigation had a highly significant impact (*p* < 0.001) on plant height, leaf number, and the dry weights of leaves, stems, and capsules. Nitrogen fertigation also exerted highly significant effects (*p* < 0.001) on plant height, leaf number, and the dry weights of leaves and capsules. The I × N interaction was statistically significant for plant height, leaf number, and all dry weight parameters but not for branch number. In contrast, the Y × I and Y × N interactions were significant for plant height and dry weight of leaves. The three-way interaction (Y × I × N) was non-significant for all traits, indicating that the two-factor interactions were consistent across years.

**Table 3:** ANOVA results for the main and interactive effects of deficit irrigation level and nitrogen fertigation rate on growth traits of sesame

Source of variance	d.f	Plant height (cm)	Branch number per plant	Leaf number per plant	Dry weight of leaves (g)	Dry weight of stem (g)	Dry weight of capsules (g)
Years (Y)	1	107.527 ^*^	7.407 ^**^	52.215 ^***^	7.782 ^*^	23.063 ^*^	34.241 ^***^
Rep. × Y	4	32.364 ^ns^	0.557 ^ns^	0.485 ^ns^	0.826 ^ns^	0.479 ^ns^	0.203 ^ns^
Irrigation (I)	2	356.233 ^***^	4.076 ^*^	1344.242 ^***^	164.762 ^***^	344.089 ^***^	469.395 ^***^
Y × I	2	95.862 ^*^	0.536 ^ns^	0.022 ^ns^	4.079 ^*^	1.845 ^ns^	0.187 ^ns^
Error (I)	8	16.636	0.699	1.893	0.768	2.283	0.361
Fertigation (N)	2	1700.134 ^***^	1.702 ^ns^	478.432 ^***^	51.052 ^***^	15.117 ^**^	149.847 ^***^
Y × N	2	120.209 ^**^	0.690 ^ns^	0.507 ^ns^	6.978 ^*^	0.610 ^ns^	6.379 ^*^
I × N	4	102.047 ^*^	0.104 ^ns^	28.086 ^***^	8.975 ^**^	117.605 ^***^	30.992 ^***^
Y × I × N	4	8.587 ^ns^	0.349 ^ns^	0.433 ^ns^	0.221 ^ns^	1.619 ^ns^	1.370 ^ns^
Error (N)	24	33.647	1.074	2.511	1.384	1.946	2.191
Seasonal effect							
2024		130.270	2.374	34.948	11.870	22.544	19.914
2025		133.092	3.115	36.914	12.629	23.851	21.507
F-test		*	**	***	*	*	***

*, **, *** = Significant at *p* < 0.05, *p* < 0.01, and *p* < 0.001, respectively. ns = non-significant

**Fig. 3:**
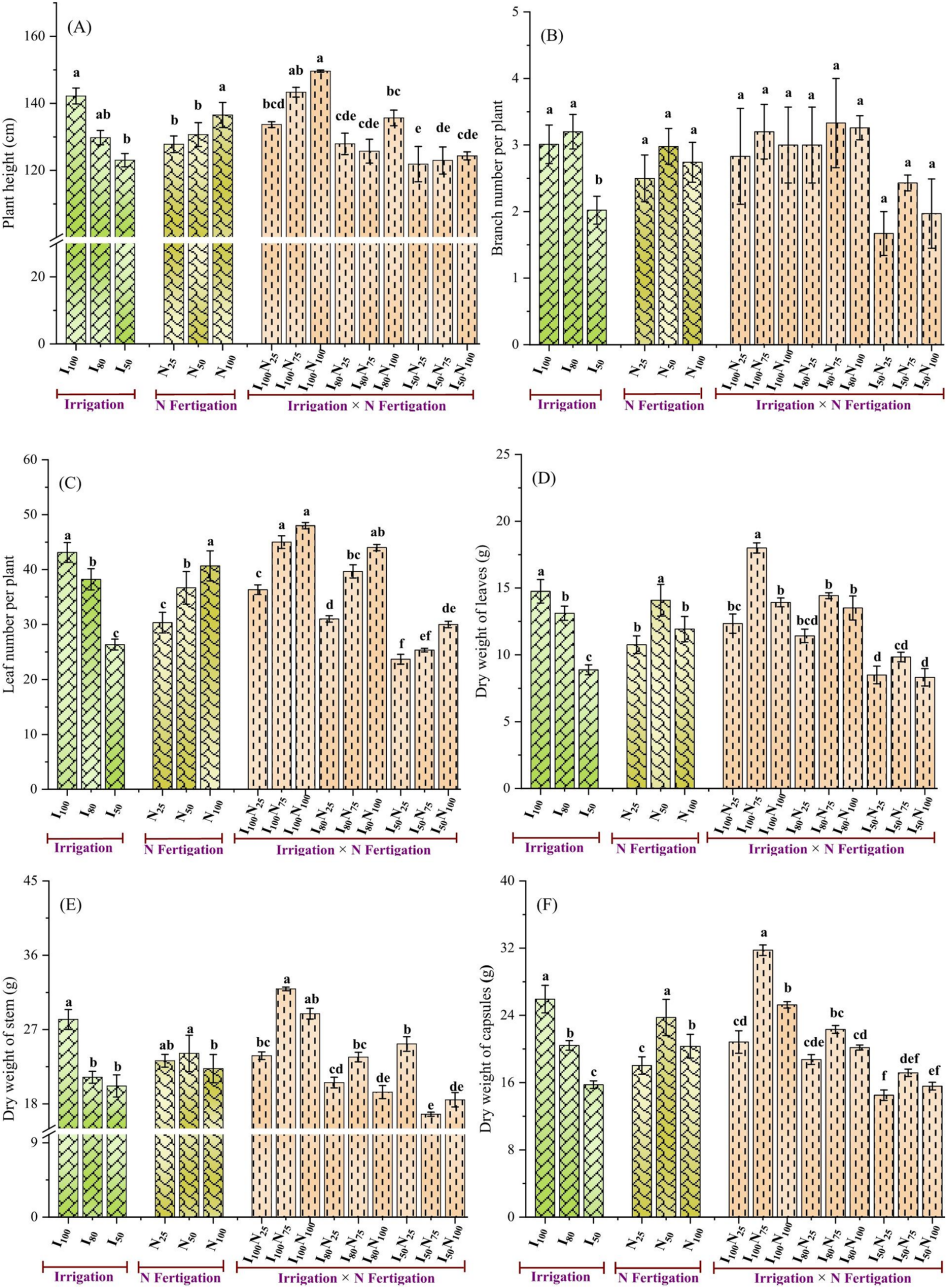
Effects of deficit irrigation levels, nitrogen fertigation rates, and their interactions on growth traits of sesame. Bars represent means ± standard deviation. Different letters above bars indicate significant differences based on Tukey’s test at *p* < 0.05

Plant height is a crucial morphological trait in sesame, directly influencing the growth and yield potential. In this regard, the effects of irrigation levels, fertigation, and their interactions on plant height of sesame were assessed. Plant height decreased progressively with increasing water deficit (Fig. 3A). The tallest plants were observed under full irrigation (142.21 ± 2.37 cm), followed by moderate deficit irrigation (129.77 ± 2.15 cm) and severe deficit irrigation (123.06 ± 1.98 cm). This trend was in line with the findings of Khasheisiuki et al. [6], who reported that water deficit reduced plant height of sesame by 22%. The decrease in plant height under drought conditions could plausibly be attributed either to reduced cell division, elongation, and differentiation caused by water deficit, leading to decreased cell turgor and growth, or to the blockage of xylem and phloem vessels, which may hinder the translocation of water, nutrients, and assimilates [37]. Similarly, the main effect of nitrogen fertigation indicated that a higher nutrient concentration (100% nitrogen level, N_100_) promoted taller plants (136.54 ± 3.73 cm) compared to the lowest dose (25%, N_25_) (127.81 ± 2.47 cm). The increase in plant height with increasing nitrogen fertigation levels is likely linked to greater nutrient availability, improved root uptake, and an increase in both the number of internodes and internodal distance [38]. The results revealed a notable interaction between irrigation levels (I) and nitrogen fertigation (N) concerning plant height (*p* < 0.05). This demonstrated that the effectiveness of nitrogen fertigation was entirely dependent on irrigation levels. A strong positive effect (*p* < 0.05) of increased nitrogen levels on plant height was observed under full irrigation (133.67 ± 0.88–149.63 ± 0.32 cm). Nonetheless, this effect was substantially reduced under 50% irrigation (121.87 ± 5.26–124.33 ± 1.20 cm) (*p* > 0.05), where plant height remained consistently low regardless of nutrient availability. These outcomes were possibly ascribed to enhanced water-nutrient co-availability, which could promote auxin- and gibberellin-mediated cell division and elongation. Under 50% irrigation, however, it is plausible that drought-induced abscisic acid accumulation may reduce stomatal conductance, root hydraulic conductivity, and nutrient transporter gene expression, while also suppressing expansion activity and gibberellin biosynthesis. Consequently, plant growth becomes primarily limited by water availability rather than nutrient supply, explaining the absence of a significant nitrogen fertigation effect under deficit irrigation. Under the studied conditions, the irrigation regime had a significant impact on branch number (*p* < 0.05). Severe water deficit (I_50_) reduced branching to 2.02 ± 0.21 branches compared to 3.20 ± 0.26 and 3.01 ± 0.29 under 80% and 100% irrigation, respectively (Fig. 3B). This reduction is probably due to reduced meristematic activity induced by water stress. However, nitrogen levels did not produce substantial effect (*p* > 0.05), suggesting that, within this range, nutrient availability was not a determinant limiting factor for branching. Furthermore, no significant interaction was detected between irrigation and nitrogen on branch number, as all treatment combinations fell within the same statistical group. Analysis of the leaf number data exhibited that both main effects and their interaction were highly significant (Fig. 3C). Full irrigation (I_100_) produced the highest leaf count (43.11 ± 1.80), whereas the lowest number (26.33 ± 1.00) was observed under I_50_. This implies that full irrigation may have supported turgor pressure for cell expansion and facilitated nutrient transport to meristematic tissues, including shoot and leaf buds. Furthermore, nitrogen fertigation exerted a strong positive effect on leaf number, where the highest nitrogen concentration (N_100_) produced significantly more leaves (40.67 ± 2.74) by 25.42 and 9.84% compared to the N_25_ and N_75_ treatments, respectively. These results are in good harmony with findings of Jadhav et al. [39]. Crucially, a significant interaction demonstrated that the benefit of increased fertigation was dependent on water availability. While nitrogen fertigation at 100% maximized leaf number substantially under the I_100_ level (48.00 ± (0.58, its effect was severely weakened under deficit irrigation (I_50_), where leaf counts remained low regardless of nutrient level (ranging from 23.67 ± 0.88–30.00 ± 0.58). This confirms that adequate irrigation is necessary for sesame plants to achieve the full vegetative growth potential under enhanced nitrogen fertigation. Furthermore, nitrogen supply and plant moisture status influenced leaf expansion and biomass production, with nitrogen deficiency augmenting the sensitivity of stomata to water stress [40]. Full irrigation consistently optimized dry matter accumulation across all organs (leaves, stems, and capsules), with values of 14.75 ± 0.88, 28.22 ± 1.20, and 25.94 ± 1.64 g, respectively (Fig. 3D–F). However, the lowest dry weights were found in water-stressed plants. This implies that water stress likely caused stomatal closure, reducing CO₂ uptake, turgor pressure, and photosynthetic efficiency, while potentially accelerating senescence, thereby minimizing carbon gain and organ biomass accumulation. Moreover, under nitrogen application, a distinct partitioning strategy emerged. A moderate nitrogen rate (N_75_) maximized the dry weight of leaves, stems, and capsules (14.08 ± 1.19, 24.11 ± 2.20, and 23.75 ± 2.15 g, respectively). Nonetheless, a low nitrogen rate (N_25_) yielded lower dry weight of leaves and capsules (10.75 ± 0.66 and 18.03 ± 1.04 g) while achieving a comparable stem dry weight (23.22 ± 0.77 g). Plants fertigated with N_75_ displayed significantly higher (*p* < 0.05) dry weight of leaves, stems, and capsules, by 15.34, 7.63, and 14.40%, respectively, than those treated with high nitrogen rate (N_100_). This suggests that the N_100_ treatment may have stimulated osmotic stress and/or nutrient antagonism (e.g., reduced K⁺ and Mg²⁺ uptake) in sesame plants, potentially leading to suppressed photosynthesis, higher assimilatory energy costs, and impaired carbon partitioning. In contrast, the moderate rate (N_75_) likely provided sufficient nitrogen for an optimal source-sink balance without inducing physiological stress, resulting in significantly higher dry weight accumulation in leaves, stems, and capsules. This positive effect is likely ascribed to the timely and frequent supply of nitrogen through drip irrigation, which met the nutritional requirements of the crop and led to maximum nutrient uptake [41]. The interaction between I_100_ and N_75_ resulted in the highest dry weights of leaves, stems, and capsules (18.00 ± 0.38, 31.92 ± 0.22 and 31.75 ± 0.63 g, respectively). Contrarily, water-stressed plants (I_50_) receiving 25%, 75%, and 100% nitrogen exhibited the lowest weights for capsules, stems, and leaves (14.50 ± 0.62, 16.75 ± 0.25 and 8.33 ± 0.65 g, respectively). This suggests that I_100_ treatment probably maintained high stomatal conductance and photosynthetic activity, while adequate nitrogen (N_75_) may have enhanced chlorophyll synthesis, Rubisco abundance, and cytokinin-mediated delay of senescence. This synergy likely improved carbon fixation, assimilate partitioning, and source-to-sink translocation, thereby maximizing biomass accumulation across vegetative and reproductive organs.

### Physiological attributes

Table 4 indicated that irrigation regimes had highly significant main effects on SPAD, chlorophyll a, chlorophyll b, and RWC (*p* < 0.001 for all). Nitrogen fertigation substantially affected SPAD, chlorophyll b, and RWC (*p* < 0.001), and chlorophyll a (*p* < 0.05). Notably, chlorophyll b exhibited the strongest synergistic response under the I × N interaction (*p* < 0.001), followed by SPAD and RWC (*p* < 0.01), and chlorophyll a (*p* < 0.05). Furthermore, the Y × I interaction was significant only for RWC (*p* < 0.05), whereas the Y × N interaction was non-significant for all parameters. The three-way interaction was significant for SPAD and RWC (*p* < 0.05 for each) but non-significant for chlorophyll a and b.

**Fig. 4:**
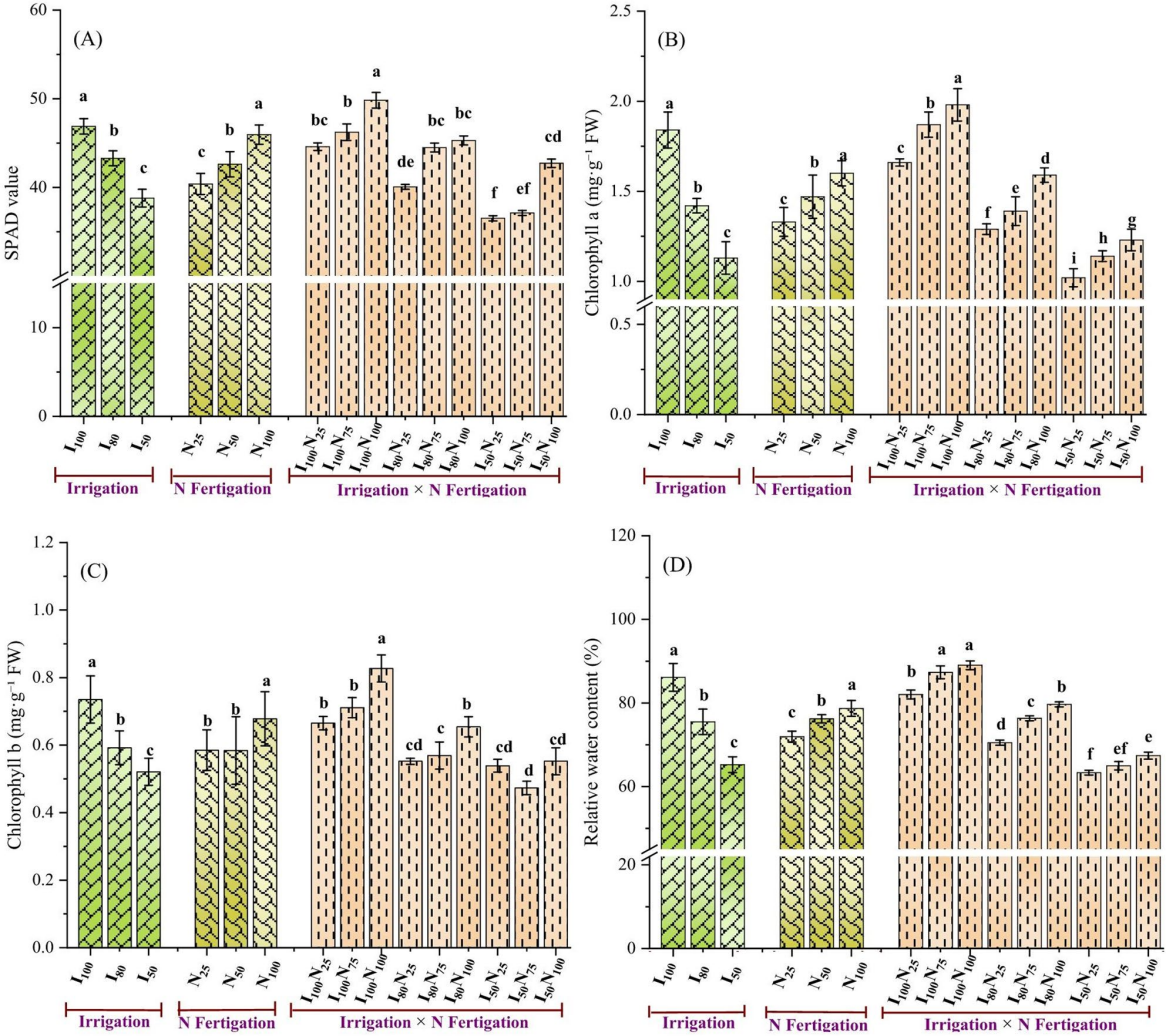
Effects of deficit irrigation levels, nitrogen fertigation rates, and their interactions on physiological traits of sesame. Bars represent means ± standard deviation. Different letters above bars indicate significant differences based on Tukey’s test at *p* < 0.05

Analysis of SPAD chlorophyll index data revealed the magnitude and dependency of treatment effects (Fig. 4A). Shifting from severe deficit irrigation (I_50_) to full irrigation (I_100_) enhanced SPAD value by 17.25% (*p* < 0.05), while increasing nitrogen fertigation from the lowest (N_25_) to highest (N_100_) concentration resulted in a 12.10% improvement. Relative to the most stressed condition (I_50_ + N_25_), the combination of full irrigation and high nitrogen rate (I_100_ + N_100_) markedly improved SPAD value by 26.69% (*p* < 0.05). This implies that water deficit may have restricted root-to-shoot nutrient transport, particularly of nitrogen and magnesium, thereby limiting chlorophyll synthesis and accelerating pigment degradation, ultimately leading to lower SPAD values. Zhao et al. [42] also reported that water stress decreases the photosynthetic rate and the production of photosynthetic products. This may, in turn, limit the ability of plants to absorb and accumulate nutrients, leading to lower chlorophyll content and, consequently, reduced SPAD values [43]. At the highest nitrogen level, reducing irrigation from full to severe deficit caused a 14.25% reduction in SPAD value, whereas under the lowest nitrogen, the same irrigation reduction led to a more pronounced decline of 18.09%. This indicates that while intensified nitrogen fertigation partially mitigated the negative impact of water deficit on chlorophyll content, it did not achieve full compensation, thus providing a quantitative assessment of the efficacy of nutrient-water interactions. Comparable results have also been reported [44]. Furthermore, the findings illustrate that plants irrigated at 100% ET_c_ had significantly higher chlorophyll a and b levels (1.84 ± 0.14 and 0.735 ± 0.07 mg·g⁻¹ FW, respectively) than water-stressed plants (irrigated at 50%) (1.13 ± 0.0.09 and 0.521 ± 0.04 mg·g⁻¹ FW) (Fig. 4B and C). The reduction in chlorophyll content under water stress was possibly associated with the oxidative damage induced by ROS, leading to the degradation of photosynthetic pigments and the disruption of chloroplast integrity, along with a stress-stimulated inhibition of chlorophyll biosynthesis [45]. Similar observations have been previously documented [7]. While the observed reduction in chlorophyll content under water stress is consistent with ROS-mediated oxidative damage reported in the literature, the lack of direct measurements of ROS and antioxidant enzyme activities represents a limitation of the present study, precluding definitive mechanistic conclusions. Future studies should therefore incorporate these biochemical analyses to establish a direct causal relationship among water deficit, ROS accumulation, antioxidant defense responses, and photosynthetic pigment degradation in sesame. Across all nitrogen fertigation levels, chlorophyll a and b exhibited distinct behavioral responses depending on the treatment conditions. Under the N_100_ treatment, chlorophyll a and b contents were 1.60 ± 0.34 and 0.678 ± 0.08 mg·g⁻¹ FW, respectively, whereas under stressed conditions at the 25% level, these values substantially declined to 1.33 ± 0.28 and 0.585 ± 0.06 mg·g⁻¹ FW (*p* < 0.05). These outcomes indicate that limited nitrogen supply may have reduced the synthesis of amino levulinic acid and glutamate, key precursors for chlorophyll biosynthesis, impaired the assembly of chlorophyll-protein complexes in the photosystems, and promoted oxidative stress, which accelerated chlorophyll degradation via enzymes such as chlorophyllase. Consequently, both chlorophyll a and b declined considerably, probably due to inhibited biosynthesis and enhanced pigment breakdown under nitrogen deficiency. Furthermore, the interaction of the I_100_ and N_100_ resulted in the highest chlorophyll a and b levels, reaching 1.98 ± 0.02 and 0.827 ± 0.04 mg·g⁻¹ FW, respectively. Contrarily, water-stressed plants under the I_50_ with N_25_ treatment showed the lowest chlorophyll a level (1.02 ± 0.04 mg·g⁻¹ FW), whilst the minimal chlorophyll b content (0.473 ± 0.02 mg·g⁻¹ FW) was recorded following the synergistic effects of I_50_ and N_75_. The observed inconsistency likely reflects differential biosynthetic sensitivity and degradation kinetics. Under water stress, severe nitrogen limitation (N_25_) could impair N-dependent enzymes (e.g., Mg-chelatase) central to chlorophyll a formation, which would preferentially reduce chlorophyll a. In contrast, moderate nitrogen supply (N_75_) may destabilize light-harvesting complexes by altering chlorophyllide a oxygenase activity or by enhancing photo-oxidative turnover, consequently lowering chlorophyll b without similarly affecting chlorophyll a. These observations suggest that drought stress negatively impacted chlorophyll contents and photosynthetic rate, substantially reducing plant growth and development [46]. Such findings were consistent with the results of growth traits, including plant height, branch number, leaf number, SPAD value, and dry weight of leaves and stems. On the other hand, the results revealed that severe water stress (I_50_) induced a significant plant water deficit, as evidenced by the lowest RWC (65.24 ± 1.88%) (Fig. 4D). Conversely, the full irrigation regime (I_100_) maximized RWC level (86.13 ± 3.33%). The decrease in RWC under water stress was probably linked to reduction in soil water potential, which constrained root water uptake to rates insufficient to meet transpirational demand [47]. Regarding nitrogen fertigation, plants fertigated with N_100_ exhibited the highest RWC (78.70 ± 0.94%), whereas the minimal level (71.96 ± 0.82%) was recorded under N_25_ treatment. This implies that N_100_ may have enhanced the synthesis of osmoprotectants (e.g., proline, glycine betaine) and upregulated aquaporin expression, which improved root hydraulic conductivity and cellular turgor maintenance, resulting in higher RWC. Conversely, nitrogen deficiency (N_25_) likely impaired osmolyte production, aquaporin activity, and root development, causing reduced water absorption and retention, thereby lowering RWC. Analysis of the interaction between irrigation levels and nitrogen fertigation treatments illustrated that the maximum RWC (89.03 ± 1.05%) was achieved under I_100_ combined with N_100_ level. Under the same irrigation regime (I_100_), no significant difference was observed between the N_100_ and N_75_ treatments (*p* > 0.05). In contrast, the lowest RWC content (63.33 ± 0.57%) was noted following the application N_25_ under water stress condition (I_50_). These findings were in agreement with those of Jan et al. [48], who reported that drought-stressed sunflower plants exhibited notable increases in osmotic adjustment parameters (e.g. RWC), which contributed to mitigating drought-induced osmotic and oxidative effects. This adjustment maintained cellular turgor and facilitated water uptake, consistent with the role of RWC as a key osmoprotectant in stabilizing proteins and membranes, thereby reducing oxidative damage [49].

**Table 4:** ANOVA results for the main and interactive effects of deficit irrigation level and nitrogen fertigation rate on physiological traits of sesame

Source of variance	d.f	SPAD values	Chlorophyll a (mg·g⁻¹ FW)	Chlorophyll b (mg·g⁻¹ FW)	Relative water content (%)
Years (Y)	1	52.412 ^***^	0.514 ^*^	0.013 ^*^	32.047 ^**^
Rep. × Y	4	2.4875 ^ns^	0.049 ^ns^	0.001 ^ns^	1.790 ^ns^
Irrigation (I)	2	141.267 ^***^	2.006 ^***^	0.209 ^***^	1963.782 ^***^
Y × I	2	0.204 ^ns^	0.105 ^ns^	0.001 ^ns^	7.880 ^*^
Error (I)	8	1.441	0.060	0.002	1.634
Fertigation (N)	2	296.460 ^***^	0.237 ^*^	0.047 ^***^	209.861 ^***^
Y × N	2	3.779 ^ns^	0.111 ^ns^	0.001 ^ns^	0.637 ^ns^
I × N	4	10.504 ^**^	0.209 ^*^	0.009 ^***^	12.168 ^**^
Y × I × N	4	4.935 ^*^	0.023 ^ns^	0.003 ^ns^	6.018 ^*^
Error (N)	24	1.747	0.063	0.002	1.841
Seasonal effect					
2024		42.004	1.385	0.599	74.859
2025		43.974	1.580	0.630	76.400
F-test		***	*	**	**

*, **, *** = Significant at *p* < 0.05, *p* < 0.01, and *p* < 0.001, respectively. ns = non-significant

### Yield and yield components

ANOVA indicated that irrigation regimes, nitrogen fertigation levels, and their interaction significantly influenced sesame yield and its components (Table 5). The main effect of irrigation was highly significant (*p* < 0.001) for all yield-related traits, confirming strong water-limited productivity. Furthermore, nitrogen fertigation substantially affected capsule number, capsule length, and seed yield (*p* < 0.001). The I × N interaction was strongly significant for capsule number and seed yield (*p* < 0.001). Among higher-order interactions, Y × I and Y × N significantly affected 1000-seed weight (*p* < 0.05), while no significant impact was detected for capsule number. The three-way interaction was not significant for all measured traits (*p* > 0.05), with the exception of capsule number.

**Table 5:** ANOVA results for the main and interactive effects of deficit irrigation level and nitrogen fertigation rate on yield and yield components of sesame

Source of variance	d.f	Capsule number per plant	Capsule length (cm)	1000-seed weight (g)	Seed yield (kg·ha⁻¹)
Years (Y)	1	20.823 ^*^	0.101 ^ns^	0.461 ^*^	332814.858 ^***^
Rep. × Y	4	8.849 ^ns^	0.013 ^ns^	0.150 ^ns^	1529.291 ^ns^
Irrigation (I)	2	4318.231 ^***^	4.022 ^***^	3.330 ^***^	581542.977 ^***^
Y × I	2	0.701 ^ns^	0.016 ^ns^	0.230 ^*^	32934.108 ^**^
Error (I)	8	3.669	0.041	0.039	3680.241
Fertigation (N)	2	1015.781 ^***^	1.383 ^***^	0.566 ^**^	67194.410 ^***^
Y × N	2	2.684 ^ns^	0.001 ^*^	1.235 ^**^	8823.743 ^ns^
I × N	4	96.712 ^***^	0.282 ^**^	0.416 ^*^	37547.806 ^***^
Y × I × N	4	30.410 ^*^	0.006 ^ns^	0.046 ^ns^	5262.617 ^ns^
Error (N)	24	8.530	0.034	0.100	2743.832
Seasonal effect					
2024		72.985	2.661	3.010	1208.302
2025		74.037	2.755	3.169	1365.315
F-test		*	ns	*	***

*, **, *** = Significant at *p* < 0.05, *p* < 0.01, and *p* < 0.001, respectively. ns = non-significant

**Fig. 5:**
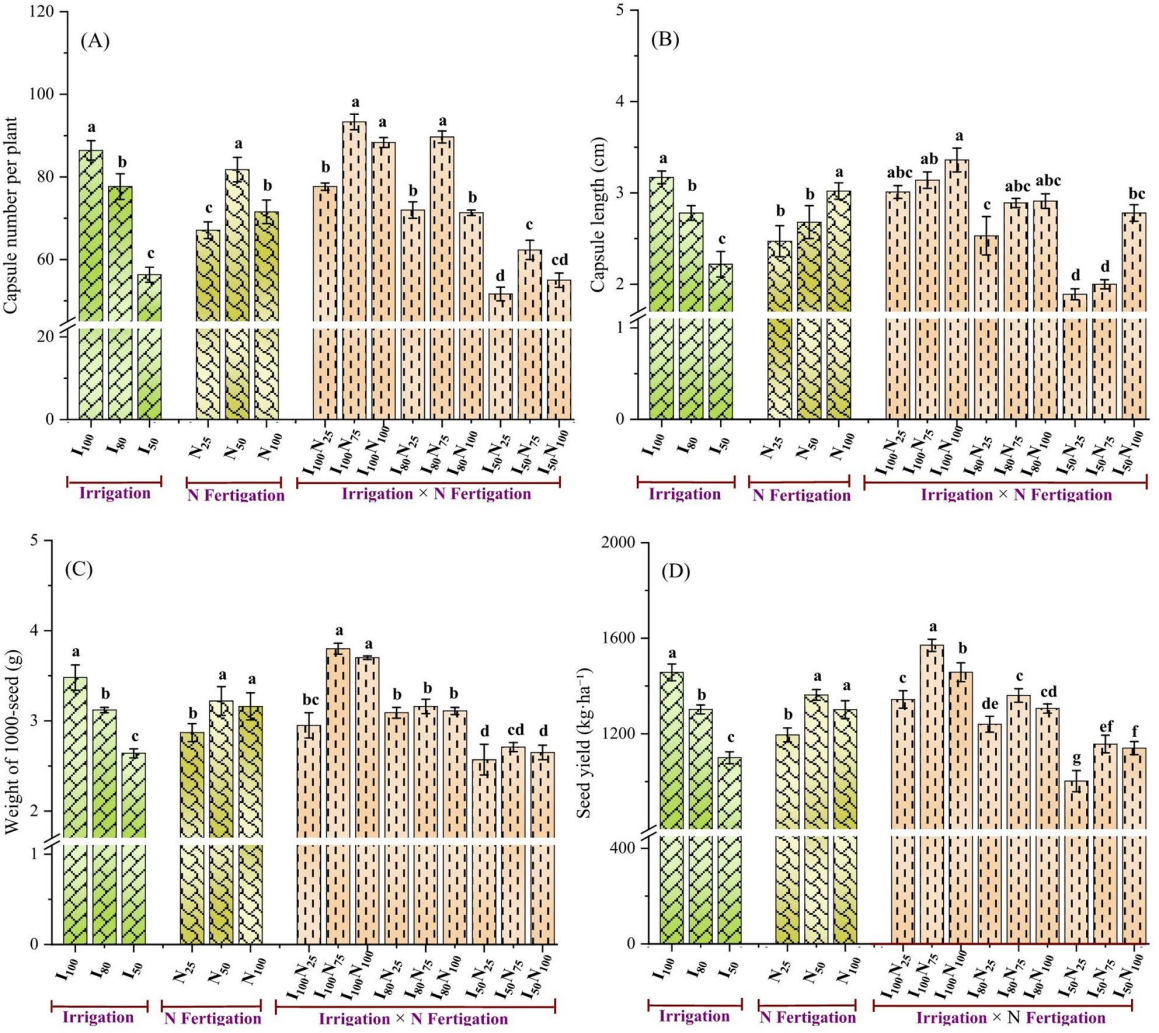
Effects of deficit irrigation levels, nitrogen fertigation rates, and their interactions on yield traits of sesame. Bars represent means ± standard deviation. Different letters above bars indicate significant differences based on Tukey’s test at *p* < 0.05

The results demonstrate that capsule number was strongly influenced by irrigation regimes, as water deficit during the critical flowering and capsule-filling stages caused substantial decreases in such trait (*p* < 0.05). The highest capsule number (86.44 ± 2.37) was documented in well-watered plants, whereas the minimum number (56.33 ± 1.84) was observed under the 50% irrigation treatment (Fig. 5A). These findings indicate that drought stress likely impaired photosynthesis and altered hormone balance (increased ABA and decreased cytokinin), potentially promoting the abscission of flowers and young capsules. Consequently, this could have led to reduced nutrient mobility and assimilate supply, which significantly lowered the mature capsule number per plant compared to well-watered plants. Full irrigation ensured a continuous supply of water and nutrients, which in turn would have minimized flower abortion and enabled a higher proportion of flowers to develop into mature capsules. Furthermore, plants fertigated with 75% nitrogen (N_75_) displayed the highest capsule number (81.78 ± 4.96). Contrarily, those receiving only 25% nitrogen (N_25_) produced a significantly lower number (67.11 ± 4.02). The N_75_ treatment remarkably increased capsule number by 12.49% (*p* < 0.05) relative to the full nitrogen. This unexpected observation may be hypothetically attributable to the excessive shoot and leaf growth following full nitrogen treatment, which was consistent with the findings of plant height, branch number, and leaf number (Fig. 3A–C). Such excessive growth likely stimulated mutual shading and intensified competition for assimilates, thereby delaying or reducing reproductive development. In contrast, N_75_ treatment may have optimized the source-sink balance by reducing excessive vegetative growth, while also improving nutrient use efficiency and the partitioning of assimilates toward reproductive structures, resulting in enhanced flower retention and capsule development. Moreover, the highest capsule number (93.33 ± 0.88) was found in well-watered plants under 100% irrigation combined with 75% nitrogen fertigation, whereas the lowest number (51.67 ± 1.67) was recorded under I_50_ and N_25_. This reduction may be attributed to a severe shortage of resources (i.e., water and nitrogen), which could limit chlorophyll synthesis, rubisco activity, and amino acid production, potentially leading to premature abscission of floral buds and capsules due to impaired carbohydrate translocation and hormonal imbalance, thereby minimizing capsule retention and final count. Regarding capsule length (Fig. 5B), full irrigation resulted in the longest capsules (3.17 ± 0.07 cm), which were statistically superior to all other treatments (*p* < 0.05), indicating this water level optimally met the physiological demands for capsule development in sesame. Water deficit treatment reduced capsule length by 5% compared to normal irrigation [50]. Notably, reducing irrigation to 80% (I_80_) decreased capsule length to 2.78 cm, suggesting that a 20% water deficit induced mild plant stress. Under such condition, plants may have redirected resources from reproduction and growth toward survival, reducing stomatal conductance to minimize water loss. This, in turn, likely limited CO₂ intake and subsequently impaired photosynthesis. Similar responses have been reported in studies of osmotic stress, where reduced stomatal conductance was closely associated with lower growth and reproductive performance under water-limited conditions [51]. The reduced availability of photosynthates (sugars and other organic compounds) may have restricted the resources allocable to developing capsules, thereby decreasing capsule length. The most pronounced reduction in capsule length (2.22 ± 0.14 cm) was observed under I_50_, probably attributable to the loss of cell turgor and the synthesis of stress hormones (e.g., abscisic acid), which further inhibited growth and may have triggered premature capsule or seed abortion [52]. Patmi et al. [53] found that the capsule length decreased from 22.7 to 14.8 mm under water stress conditions. Remarkably, the N_100_ treatment increased capsule length by 18.21% and 11.29% compared to plants fertigated with the 25% and 75% nitrogen, respectively (*p* < 0.05). This reveals that N_100_ may have delayed leaf senescence and preserving sink strength directed toward reproductive structures. Moreover, the synergistic application of I_100_ and N_100_ maximized capsule length (3.36 ± 0.13 cm), whereas the shortest capsules (1.89 ± 0.06 cm) were observed under severe deficit irrigation (I_50_) and 25% nitrogen fertigation (N_25_), a trend similar to that reported by Chauhan et al. [54]. The 1000-seed weight, a key component of seed yield and a reliable parameter of seed plumpness and physiological maturity, was significantly influenced by different irrigation regimes (Fig. 5C). Relative to I_50_ and I_80_, the I_100_ treatment significantly increased the 1000-seed weight by 24.14% and 10.34% (*p* < 0.05). These findings indicate that full irrigation (100% ET_c_) met the water requirements of sesame during the seed filling and maturation stages, which could have enhanced the translocation of assimilates (e.g., starch and protein) to seeds and increasing seed weight. Moreover, the 1000-seed weight augmented significantly from 2.87 ± 0.10 g at a 25% N_25_ to 3.22 ± 0.16 g at 75% (N_75_), indicating that the lower rate was insufficient for optimal seed development. However, no further significant increase was observed at 100% (3.16 ± 0.15 g; *p* > 0.05), suggesting that N_75_ met the nutritional requirements for maximal seed dry matter accumulation, with no additional benefit beyond this threshold. The maximum 1000-seed weight (3.80 ± 0.06 g) was recorded for well-watered plants fertigated at 75%, closely followed by well-watered plants with 100% nitrogen fertigation (3.70 ± 0.02 g). Nevertheless, the minimum weight (2.57 ± 0.17 g) was recorded under the interaction of I_50_ and N_25_. The synergistic effect of water and nutrient management, achieved through fertigation, possibly enhanced nutrient assimilation by ensuring nutrient availability during critical developmental stages, thereby reducing dependency of plants on soil-bound nutrients [55]. These outcomes illustrate that water deficit may have impaired photosynthetic assimilation, sugar metabolism, and nutrient translocation to the seeds, resulting in reduced seed weight, consistent with the findings of Ucan et al. [56]. Figure 5D shows that I_100_ regime maximized the seed yield (1457.30 ± 35.16 kg·ha⁻¹), followed by plants irrigated under the I_80_ (1302.21 ± 18.83 kg·ha⁻¹) and I_50_ (1099.84 ± 25.08 kg·ha⁻¹) treatments (*p* < 0.05). These observations indicate that I_100_ maintained soil moisture near the field capacity for sesame throughout the growing season, particularly during phenologically sensitive stages (flowering, pollination, and seed formation). This water regime likely also improved nutrient uptake and translocation and alleviated physiological stress, thereby optimizing reproductive metabolic processes (ovule fertilization, embryogenesis, seed filling) and significantly increasing yield. Comparable observations have been reported in literature [5, 57]. The ANOVA revealed that seed yield was significantly (p *<* 0.001) affected by the nitrogen fertigation treatments. Plants receiving 25% nitrogen (N_25_) produced the lowest seed yield (1195.31 ± 29.28 kg·ha⁻¹). Surprisingly, the maximum yield was observed under the 75% nitrogen (1362.78 ± 22.62 kg. ha^− 1^), which was higher (by 4.51%) than that of plants fertigated at 100% level. This variation in seed yield was positively associated with results of capsule number per plant and 1000-seed weight (Fig. 5A and C). This can be explained by the fact that the 100% dose (N_100_) likely induced excessive vegetative growth, potentially leading to greater allocation of photosynthates toward leaves and stems rather than reproductive structures (capsules and seeds). Additionally, this treatment may have stimulated ionic imbalances (e.g., suppressed uptake of potassium, calcium, or magnesium) osmotic stress, ammonium buildup in the rhizosphere, and delayed maturity, thereby reducing seed yield. Remarkably, the interaction between irrigation and nitrogen application significantly influenced seed yield (*p* < 0.05). Maximum yield (1570.51 ± 25.31 kg·ha⁻¹) was recorded under full irrigation and 75% nitrogen fertigation, while the combination of severe water deficit and 25% nitrogen fertigation resulted in the lowest yield (1002.10 ± 44.43 kg·ha⁻¹). Similarly, El-Tantawy et al. [58] found that water stress reduced sesame yield, with the optimal irrigation amount for sesame ranging from 4367 to 4728 m^3^.ha^− 1^.

### Chemical analysis

ANOVA revealed differential effects of irrigation and nitrogen fertigation on sesame quality (Table 6). The main effect of irrigation was highly significant for oil content and nitrogen content (*p* < 0.001). Nitrogen fertigation also remarkably affected both parameters (*p* < 0.001). Moreover, significant synergistic effects were observed for oil content (*p* < 0.05) and nitrogen content (*p* < 0.01). Notably, the Y × I and Y × N interactions were not significant for either parameter. The three-way interaction was highly significant for nitrogen content (*p* < 0.001), whereas no significant effect was recorded for oil content under this interaction.

**Table 6:** ANOVA results for the main and interactive effects of deficit irrigation level and nitrogen fertigation rate on chemical attributes and crop water productivity of sesame

Source of variance	d.f	Oil content (%)	Nitrogen content (%)	CWP (kg·m⁻³)
Years (Y)	1	47.790 ^**^	1.084 ^**^	0.001 ^*^
Rep. × Y	4	0.788 ^ns^	0.132 ^ns^	0.002 ^ns^
Irrigation (I)	2	927.431 ^***^	4.328 ^***^	0.120 ^***^
Y × I	2	0.046 ^ns^	0.002 ^ns^	0.006 ^**^
Error (I)	8	3.392	0.064	0.001
Fertigation (N)	2	74.884 ^***^	0.769 ^***^	0.004 ^**^
Y × N	2	1.604 ^ns^	0.108 ^ns^	0.001 ^ns^
I × N	4	6.303 ^*^	1.204 ^**^	0.003 ^**^
Y × I × N	4	0.785 ^ns^	2.157 ^***^	0.001 ^ns^
Error (N)	24	2.101	0.080	0.001
Seasonal effect				
2024		49.148	2.823	0.297
2025		51.030	3.126	0.306
F-test		**	**	*

*, **, *** = Significant at *p* < 0.05, *p* < 0.01, and *p* < 0.001, respectively. ns = non-significant

**Fig. 6:**
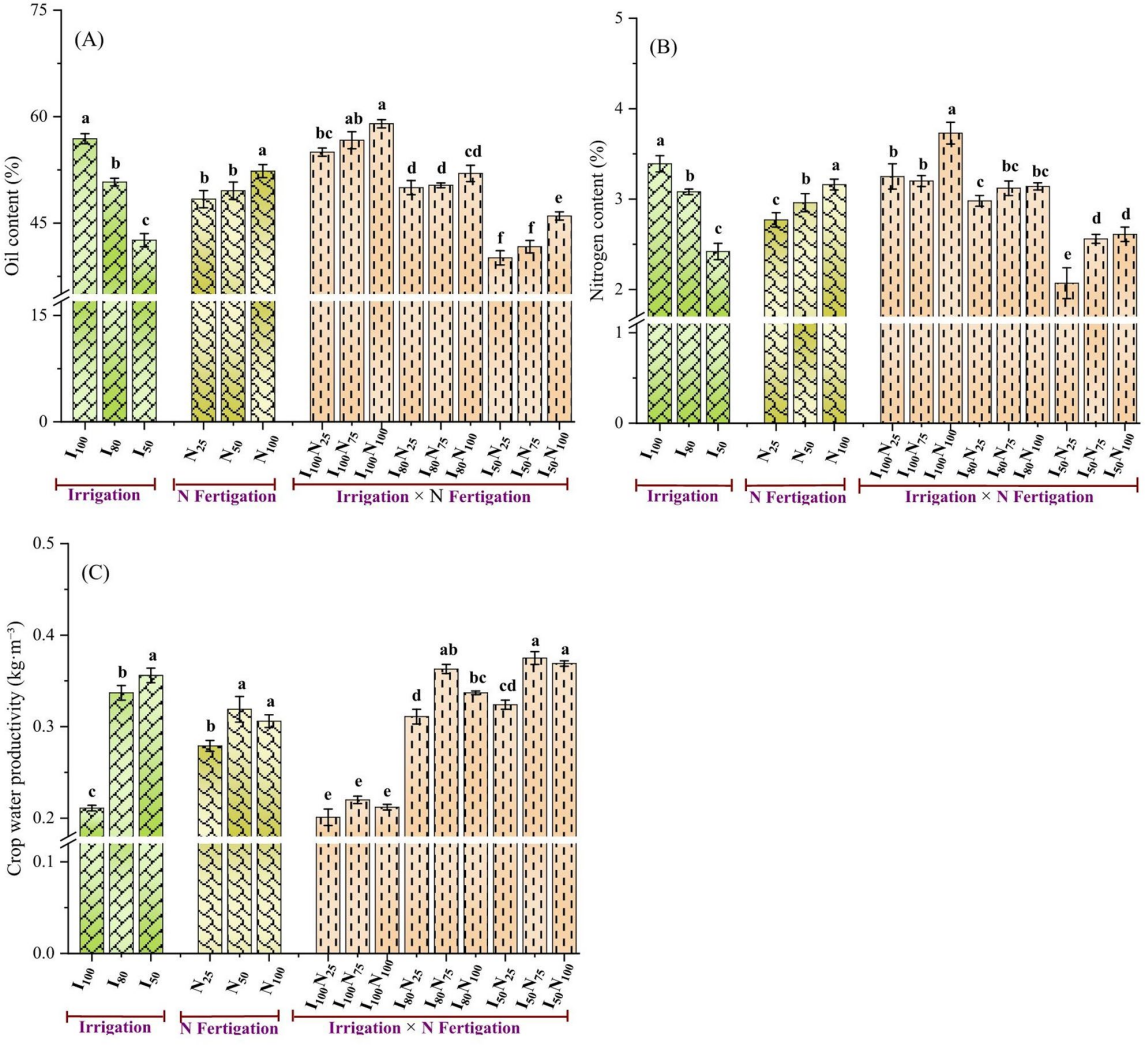
Effects of deficit irrigation levels, nitrogen fertigation rates, and their interactions on quality traits and crop water productivity of sesame. Bars represent means ± standard deviation. Different letters above bars indicate significant differences based on Tukey’s test at *p* < 0.05

Concerning oil content (Fig. 6A), the 100% irrigation regime increased sesame oil content (56.89 ± 0.71%), whereas the lowest content was noted in water-stressed plants (42.59 ± 0.93%). This implies that water stress may have induced lipid peroxidation, which damaged membrane lipids, precursors to seed oils [59]. Water stress may also suppress lipid biosynthesis and enhance lipolytic and peroxidative activities, ultimately reducing oil content. Additionally, negative effects of water stress on sesame oil content have been highlighted previously [60, 61]. The observed reduction in oil content under severe drought represents a substantial and economically significant loss. This impacts marketable yield and processor profitability. Consequently, the marked sensitivity of oil accumulation to water deficit underscores the need for targeted irrigation management or the breeding of drought-tolerant, high-oil genotypes, as grain yield alone may not fully capture the economic consequences of water stress in sesame. Moreover, fully irrigated plants (100% ET_c_) showed the highest nitrogen content (3.39 ± 0.09%) (Fig. 6B), which was significantly greater by 9.14 and 28.61% than in the I_80_ and I_50_ treatments, respectively (*p* < 0.05). This phenomenon was likely attributable to enhanced root growth and metabolic activity, which probably increased the solubility and mobility of soil nitrates and improved the efficiency of transpiration-driven mass flow of nitrogen toward the root system. Consequently, nitrogen uptake and assimilation were augmented under full irrigation treatment. These improvements may have subsequently enhanced metabolic processes, such as amino acid biosynthesis and protein formation, within the seeds, ultimately leading to an increase in the nitrogen content of sesame seeds. These findings were in accordance with those reported by Fazeli et al. [62], who observed that water stress reduced protein synthesis in sesame leaves and suggested that water stress may generate ROS, thereby inhibiting protein synthesis or causing protein denaturation. The positive interaction between water availability and nitrogen supply in enhancing sesame oil and protein content is consistent with findings reported for other oilseed crops. For instance, Alipour Babadi et al. [21] showed that optimized nutrient supply significantly improves leaf area and photosynthetic efficiency, both of which play critical roles in the synthesis and accumulation of lipids and proteins during the seed-filling stage. Sesame plants subjected to nitrogen fertigation exhibited notable responses in oil and nitrogen content across different nitrogen fertigation rates (100% ˃ 75% ˃ 25%). The findings revealed significant variation in oil and nitrogen content, with the maximum values (52.33 ± 0.92% for oil and 3.16 ± 0.06% for nitrogen) achieved under the application of N_100_, compared to N_25_ treatment, which resulted in the minimal contents (48.37 ± 1.21 and 2.77 ± 0.08%, respectively). Comparable results have been documented in literature [63]. Moreover, different irrigation levels and nitrogen fertigation doses led to observable variations in oil and nitrogen content of sesame. The highest oil and nitrogen contents (59.00 ± 0.58% and 3.73 ± 0.12%, respectively) were recorded under full irrigation (I_100_) combined with N_100_ level, in contrast to the interaction of deficit irrigation (I_50_) with N_25_ treatment, which yielded the lowest contents. Although the highest oil content (59.00 ± 0.58%) was observed under full irrigation with 100% nitrogen application (I_100_N_100_), this value did not differ significantly from the oil content recorded under I_100_N_75_ (56.67 ± 1.20%). More critically, reducing the nitrogen rate from 100% to 75% under full irrigation led to a significant increase in seed yield (from 1457.68 ± 39.41 to 1570.51 ± 25.31 kg·ha⁻¹). Consequently, the oil yield per hectare was higher for I_100_N_75_ (890.01 kg oil·ha⁻¹) than for I_100_N_100_ (860.03 kg oil·ha⁻¹). This indicates that applying an additional 25% nitrogen under full irrigation did not provide a statistically meaningful increase in oil content but rather resulted in a measurable reduction in seed and oil yield. Therefore, from an economic and resource efficiency perspective, the I_100_N_75_ treatment is superior as it achieves higher oil yield per unit area while reducing nitrogen input by 25% and lowering production costs. Thus, the I_100_N_75_ treatment represents a more efficient balance between oil content and productivity, achieving higher yields with lower nitrogen input, thereby aligning with the goals of sustainable intensification.

### Crop water productivity

Table 6 shows that CWP was significantly influenced by irrigation regime, nitrogen fertigation, and their interaction. Irrigation had a highly significant main effect (*p* < 0.001), dominating CWP determination, followed by a smaller but significant effect of nitrogen fertigation (*p* < 0.01). The two-way interaction I × N was significant (*p* < 0.01), indicating a synergistic effect of irrigation and fertigation on CWP. The observed improvements in sesame growth and nutrient uptake under optimized irrigation and nitrogen levels may be linked to enhanced rhizosphere dynamics. Alipourbabadi et al. [64] reported that advanced nutrient management can reorganize root-associated enzyme hotspots and expand biologically active zones in the rhizosphere, thereby promoting tighter root-microbe feedbacks and improving root architecture for better nutrient foraging. These rhizosphere-mediated responses may partially explain the synergistic effects of irrigation and nitrogen fertigation on CWP through enhanced water and nutrient acquisition efficiency. The Y × N and Y × I × N interactions were non-significant, confirming the temporal consistency of fertigation effects and their interaction with irrigation across both years.

The results demonstrate a progressive reduction in irrigation from 100% to 50% of ET_c_ led to a statistically significant improvement in CWP, with corresponding values increasing from 0.211 ± 0.003 to 0.356 ± 0.008 kg·m⁻^3^ (Fig. 6C). This suggests that sesame plants subjected to severe drought stress displayed higher CWP than those under full irrigation conditions. These outcomes were consistent with what was previously reported [65, 66], further supporting plant water deficit intensified as soil moisture availability decreased. This phenomenon indicates that the reduction in seed yield under deficit irrigation (I_50_) was proportionally smaller than the 50% reduction in water input, resulting in an increased yield-to-water quotient. Physiologically, it is plausible that water stress induces partial stomatal closure, which would reduce transpiration to a greater extent than photosynthesis, thereby improving intrinsic water-use efficiency. Although reducing irrigation from 100% to 50% of ET_c_ significantly increased CWP by 40.73%, this improvement was accompanied by a substantial reduction (24.53%) in seed yield (from 1457.3 to 1099.84 kg·ha⁻¹). This inverse relationship highlights a fundamental balance between water-use efficiency and land productivity. Consequently, the increased CWP under severe deficit irrigation (I_50_) does not necessarily indicate an economically or agronomically optimal strategy. While such an approach may be rational in water-scarce environments where irrigation water is the primary limiting factor, it is less justifiable when land value, fixed production costs, or yield stability are prioritized. Therefore, CWP should be interpreted alongside total yield rather than as a standalone indicator of crop performance. Concurrently, nitrogen fertigation concentration exerted a significant influence, with the N_75_ treatment yielding the highest CWP (0.319 ± 0.006 kg·m⁻³), followed by N_100_ (0.306 ± 0.007 kg·m⁻³) and N_25_ (0.279 ± 0.006 kg·m⁻³) treatments. This finding can be attributed to the likelihood that nitrogen fertigation with 75% of the nitrogen requirement provided an optimal nitrogen supply, potentially promoting root proliferation, canopy development, and stomatal regulation. In turn, this may have enabled maximal photosynthetic rates per unit of water lost, while avoiding the vegetative overgrowth or osmotic stress associated with higher nitrogen levels. Such balanced nitrogen nutrition likely enhanced both the harvest index and water-use efficiency, ultimately leading to the highest CWP. Critically, the highest CWP (0.375 ± 0.007 kg·m⁻³) was recorded under stressed conditions (i.e., 50% irrigation) combined with 75% nitrogen fertigation (N_75_). This value was statistically comparable to that observed under the same irrigation level (50%) with 100% fertigation (0.369 ± 0.002 kg·m⁻³) and was significantly superior to all other treatments. These results are in agreement with those obtained by Tura and Tolossa [67]. Conversely, plants irrigated at 100% ET_c_ (I_100_) and receiving only 25% nitrogen (N_25_) exhibited the lowest CWP (0.201 ± 0.009 kg·m⁻³), This implied that suboptimal nitrogen likely restricted the ability of plants to utilize fully applied water, resulting in unproductive water losses through evaporation, thereby lowering CWP. The maximum CWP recorded under I_50_N_75_ (0.375 kg.m⁻^3^) came at the cost of a 26.31% yield reduction (413.26 kg·ha⁻¹) compared to I_100_N_75_, despite a 50% saving in irrigation water. This context dependency is critical, as such a strategy may be acceptable in water-scarce regions where water is the primary limiting factor, whereas not where land productivity or yield stability is prioritized. Given that no economic analysis was conducted, future studies are recommended to establish water-saving thresholds based on local water costs and land values to determine the feasibility of deficit irrigation strategies.

#### Radar plot analysis

**Fig. 7:**
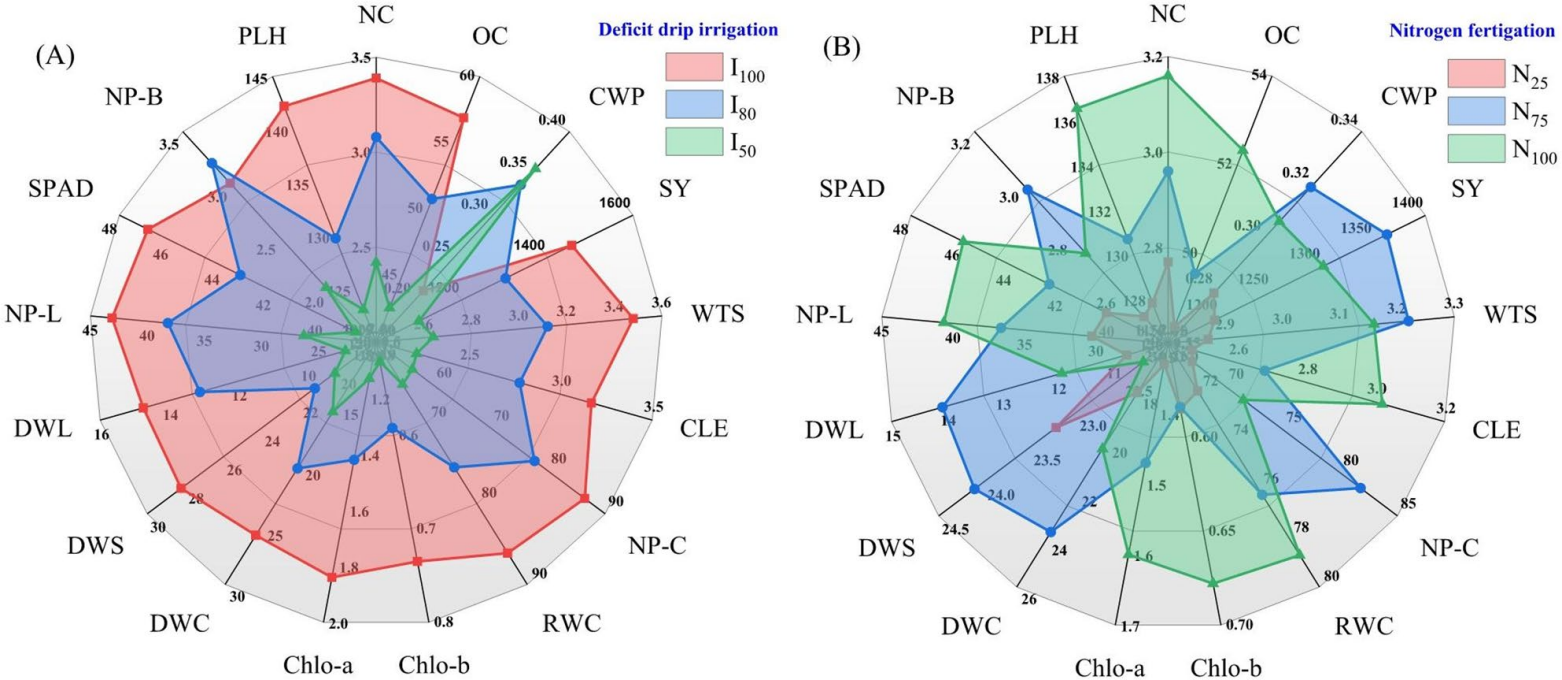
Radar plots exhibiting the effects of two treatments, deficit drip irrigation regimes (**A**) and nitrogen fertigation rates (**B**) on morpho-physiological, agronomic, and quality properties of sesame. PHL = plant height, NP-B = branch number, NP-L = leaf number, DWS = dry weight of stem, DWL = dry weight of leaves, DWC = dry weight of capsule, Chlo-a = chlorophyll a, Chlo-b = chlorophyll b, RWC = relative water content, NP-C = capsule number, CLE = capsule length, WTS = weight of 1000-seed, SY = seed yield, NC = nitrogen content, OC = oil content. CWP = crop water productivity

Radar plots are an effective tool for comparative multivariate analysis, enabling an intuitive way to visualize interdependencies across different measured traits for each treatment, with axes radiating from the center [68, 69]. The analysis displayed a clear hierarchy in how deficit drip irrigation regimes and nitrogen fertigation rates affected morpho-physiological, agronomic, and quality properties of sesame (Fig. 7A and B). Under full irrigation (I_100_, red line), a dominant contribution (approximately 90–100% of the total observed effect) was observed for growth, photosynthetic pigments, yield components, and seed chemical characteristics (Fig. 7A). Biologically, this profile indicates that providing the full crop water requirement throughout the growing season may have alleviated hydraulic limitations, thereby potentially sustaining stomatal conductance, maximizing CO₂ assimilation, and supporting nutrient translocation to developing sinks. The increase in biomass, chlorophyll content, and reproductive output confirms that water availability was non-limiting for primary metabolism. This treatment, however, contributed the least to CWP, suggesting that sesame plants reached physiological saturation, beyond which additional water did not proportionally enhance carbon assimilation or biomass accumulation, thereby reducing water productivity. In contrast, moderate deficit irrigation (I_80_, blue line) contributed intermediately (28–76%) across most traits, with pronounced effects on capsule number, leaf number, nitrogen content, and CWP, and maximal contribution to branch number. This regime likely induced mild abiotic stress, which could have triggered compensatory mechanisms such as increased root-to-shoot ratio, altered phytohormonal signaling (e.g., abscisic acid-mediated stomatal regulation), and remobilization of assimilates to reproductive structures, thereby optimizing branch and capsule formation without severe photosynthetic penalties. Conversely, the water stress treatment (I_50_, green line) exerted the lowest contributions to most parameters, yet it was the primary driver of CWP. This implied that limited water availability possibly led to overproduction of ROS, which may have resulted in oxidative damage to chloroplasts, chlorophyll degradation, suppressed cell expansion, impaired nutrient uptake and xylem transport, and dysregulation of secondary metabolism. Consequently, these effects resulted in minimized growth, yield, and nutritional quality attributes of sesame. Regarding nitrogen fertigation (Fig. 7B), the N_100_ treatment (green line) had the highest contribution plant height, SPAD value, leaf number, chlorophyll a and b, RWC, capsule length, oil content, and nitrogen content. However, for branch number, dry weight of leaves, stems and capsules, capsule number, 1000-seed weight, seed yield, and CWP, its contribution ranged from around 33–81%. This suggests that excessive nitrogen availability (N_100_) favored vegetative vigor and leaf-level physiological traits, but at the expense of reproductive allocation, possibly due to prolonged vegetative growth, reduced source-sink translocation of carbohydrates to seeds, or altered hormonal balance (e.g., lower cytokinin-to-auxin ratios in reproductive tissues). Conversely, a moderate reduction to 75% nitrogen (blue line) optimized biomass partitioning toward reproductive organs, improved water-use efficiency, reflecting a shift from nitrogen-driven vegetative expansion to carbon allocation for seed filling. The N_25_ rate (red line) showed the lowest values, remaining below 54% for all assessed traits. This implied that insufficient nitrogen availability may have limited key physiological and morphological processes, including photosynthesis, cell expansion and division, biomass accumulation, reproductive development, and overall water use efficiency. Consequently, the N_25_ treatment led to nitrogen deficiency stress, which probably impaired source-sink relationships and resulted in significantly reduced seed yield and crop water productivity. Overall, the radar visualization demonstrates that the full irrigation regime combined with N_75_ rate consistently optimized sesame productivity.

#### Principal component analysis

**Fig. 8:**
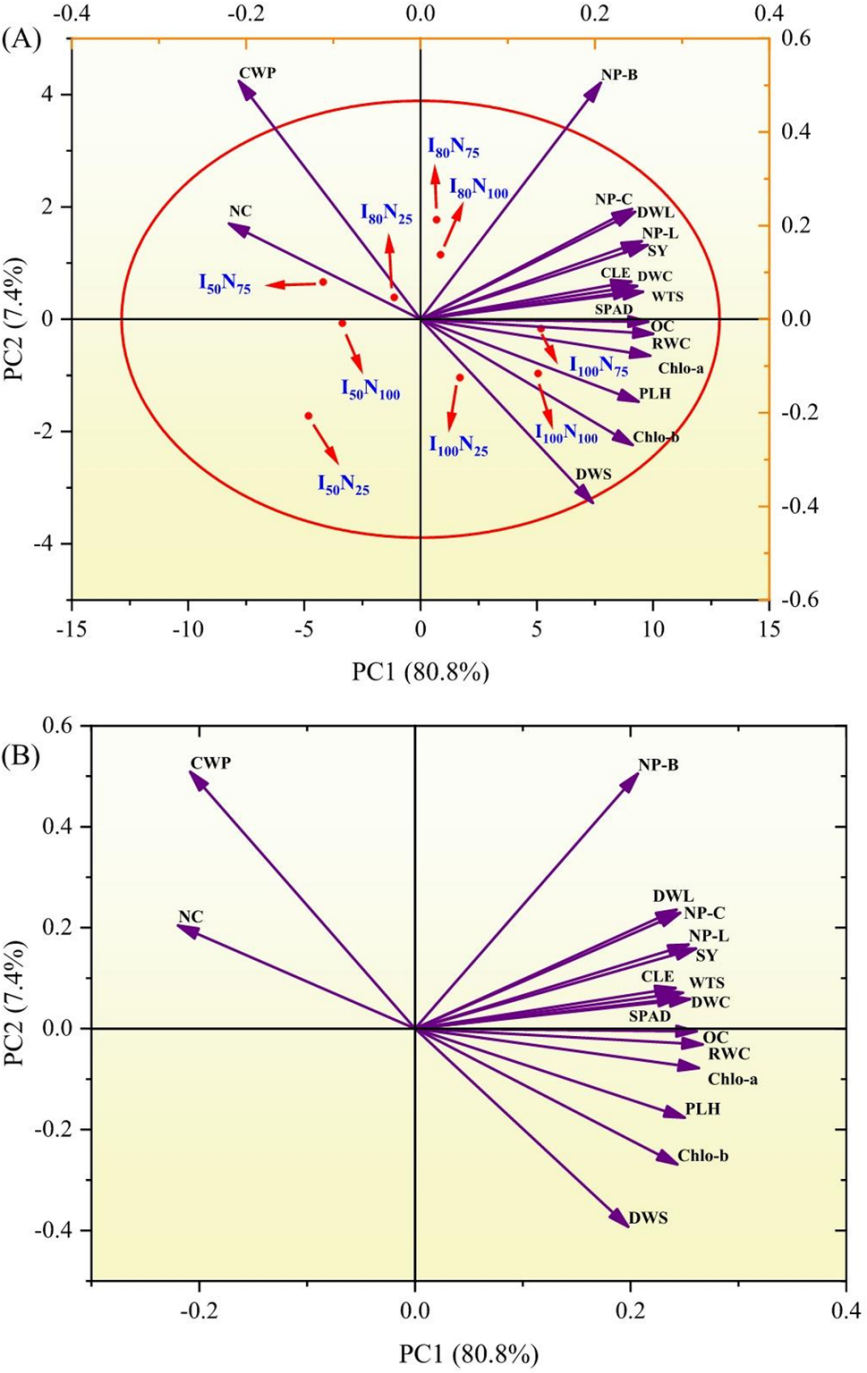
PCA (**A**), and loading plot (**B**) for morpho-physiological, agronomic, and quality properties of sesame under different deficit drip irrigation regimes and nitrogen fertigation rates. Ellipses represent the 95% confidence region for the centroid of each treatment group. PHL = plant height, NP-B = branch number, NP-L = leaf number, DWS = dry weight of stem, DWL = dry weight of leaves, DWC = dry weight of capsule, Chlo-a = chlorophyll a, Chlo-b = chlorophyll b, RWC = relative water content, NP-C = capsule number, CLE = capsule length, WTS = weight of 1000-seed, SY = seed yield, NC = nitrogen content, OC = oil content. CWP = crop water productivity

To simplify the interpretation of the synergistic effects of deficit drip irrigation levels and nitrogen fertigation rates on the morpho-physiological, agronomic, and quality attributes of sesame, PCA was performed (Fig. 8A). The PCA plot effectively illustrated the clustering of irrigation regimes and nitrogen fertigation levels according to their similarities, thereby decreasing the dimensionality of the dataset and elucidating underlying relationships between the measured traits [70, 71]. The analysis exhibited that PC1 explained 80.80% of variance, and PC2 explained an additional 7.40%, cumulatively accounting for 88.20%. The loading plot (Fig. 8B) shows that PC1 was driven primarily by positive loadings from most agronomic and physiological traits, particularly RWC (0.267), oil content (0.261), seed yield (0.260), chlorophyll a (0.263), and 1000-seed weight (0.255), whereas nitrogen content (-0.220) and CWP (-0.208) loaded negatively. In contrast, PC2 was strongly and positively influenced by branch number (0.505) and CWP (0.509), but negatively affected by stem dry weight (-0.393) and chlorophyll b (-0.269). These loading patterns indicate that clustering of samples along PC1 was mainly characterized by physiological, yield-related, and quality traits, while separation along PC2 was predominantly governed by branching, water use efficiency, and vegetative stem growth. Accordingly, these findings provide a clear interpretation of the sample grouping observed in the corresponding score plot (Fig. 8A). The first group, located in the lower right-hand quadrant with plant height, chlorophyll a and b, RWC, dry weight of stem, and oil content, comprised treatments that mainly had full irrigation (I_100_) with different nitrogen fertigation rates. This suggests that sufficient water and nitrogen fertigation may have synergistically enhanced mesophyll activity, photosynthetic efficiency, and biomass allocation, ultimately leading to improved oil production. A distinct group, situated in the positive sides of both components, was formed by treatments that combined moderate irrigation level (I_80_) with moderate-to-high nitrogen rates (N_75_ and N_100_). This synergistic effect was strongly linked to a full spectrum of traits, including vegetative growth parameters (dry weight of leaves, leaf number, and dry weight of capsules) and all major yield components (1000-seed weight, capsule number, capsule length, and seed yield). These observations may be attributable to an optimized balance between water and nitrogen availability, which could have enhanced root function, facilitated nutrient uptake, and alleviated physiological stress. Such improvements, in turn, likely promoted efficient biomass allocation and translocation of photo-assimilates from source to sink, thereby channeling resources toward reproductive growth and all yield-related components. The third group, containing I_80_N_25_ and I_50_N_75_, was positioned in the upper left-hand quadrant with CWP and nitrogen content. This placement implies that these treatments achieved efficient water use for yield, Conversely, under water stress (I_50_), the treatments that received 25% and 100% nitrogen (N_25_ and N_100_) grouped independently, displaying no associations with any measured trait. This finding demonstrates that full irrigation was necessary to elicit distinct growth, physiological, and yield responses in sesame.

### Pearson’s correlation analysis

**Fig. 9:**
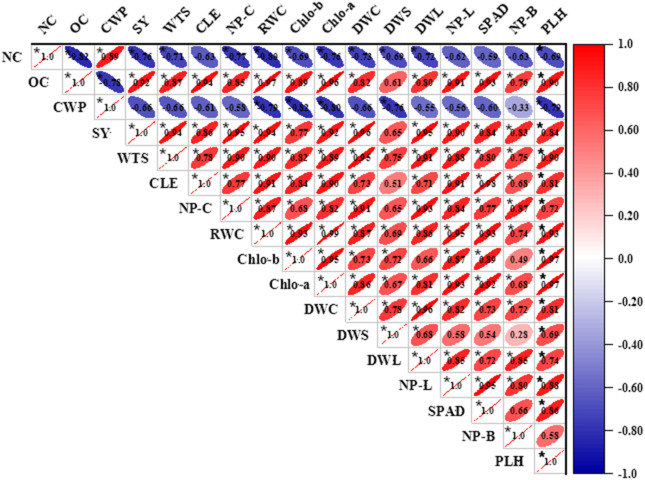
Pearson’s correlation analysis for morpho-physiological, agronomic, and quality properties of sesame under different deficit drip irrigation regimes and nitrogen fertigation rates. Correlations were calculated using the mean values of each parameter across all treatment combinations (*n* = 9). “*” represents significant at *p* < 0.05. PHL = plant height, NP-B = branch number, NP-L = leaf number, DWS = dry weight of stem, DWL = dry weight of leaves, DWC = dry weight of capsule, Chlo-a = chlorophyll a, Chlo-b = chlorophyll b, RWC = relative water content, NP-C = capsule number, CLE = capsule length, WTS = weight of 1000-seed, SY = seed yield, NC = nitrogen content, OC = oil content. CWP = crop water productivity

Pearson’s correlation analysis was conducted to elucidate the interrelationships among morpho-physiological, agronomic, and quality attributes of sesame under varying deficit drip irrigation regimes and nitrogen fertigation rates (Fig. 9). Plant height exhibited strong positive associations with the leaf number (*r* = 0.88), leaf dry weight (*r* = 0.74), capsule dry weight (*r* = 0.81), chlorophyll a (*r* = 0.97), chlorophyll b (*r* = 0.97), and RWC (*r* = 0.93), indicating that height serves as an integrated proxy for photosynthetic capacity, biomass partitioning, and plant water status. The near-perfect correlations with chlorophyll pigments reflect enhanced light capture and carbon assimilation in taller plants, supporting greater leaf production and dry matter accumulation in both vegetative and reproductive structures. Furthermore, the strong positive association with RWC suggests that sustained cell turgor and favorable plant water relations are critical for vertical stem elongation, as turgor-driven cell expansion fundamentally governs internode extension. The improvement in chlorophyll pigments (Chlo-a and Chlo-b) and RWC was responsible for the increase of 68–87% in capsule number, 84–91% in capsule length, 82–90% in 1000-seed weight, and 77–94% in seed yield. These outcomes were probably attributed to the synergistic enhancement of photosynthetic efficiency and cellular hydration status. Specifically, the elevated levels of chlorophyll pigments may have augmented light absorption and energy conversion within photosystems I and II, potentially promoting the synthesis of photoassimilates and enhancing carbohydrate biosynthesis required for reproductive development [72]. Simultaneously, the increased RWC may have maintained optimal stomatal conductance and nutrient translocation, prevented drought-induced floral abortion while facilitated capsule elongation and seed filling. The enhancement in these physiological parameters likely optimized source-sink dynamics, resulting in higher capsule retention and greater seed weight through enhanced assimilate partitioning, thereby contributing to increased yield-related traits of sesame. Further, chlorophyll pigments and RWC were negatively correlated with nitrogen content (*r* = -0.69–0.80) and CWP (*r* = -0.79–0.82), but displayed a positive relationship with oil content (*r* = 0.89–0.97). This was possibly due to the downregulation of chlorophyll synthesis and reduction RWC via partial stomatal closure, which lowered CWP. However, these conditions may have shifted metabolism from protein-rich vegetative biomass to the synthesis of storage lipids, thereby increasing oil content. Furthermore, the observed negative correlation between chlorophyll pigments and NC was likely attributable to a dilution effect and altered nitrogen partitioning, wherein assimilated nitrogen was preferentially allocated to oil synthesis and carbon-rich reserves, thereby reducing seed nitrogen concentration. Additionally, nitrogen was retained predominantly in vegetative tissues (leaves and stems) rather than being remobilized to seeds. In summary, the statistical analysis indicated that the growth and physiological attributes of sesame were substantially interrelated with its yield components and chemical properties.

## Heatmap analysis

**Fig. 10:**
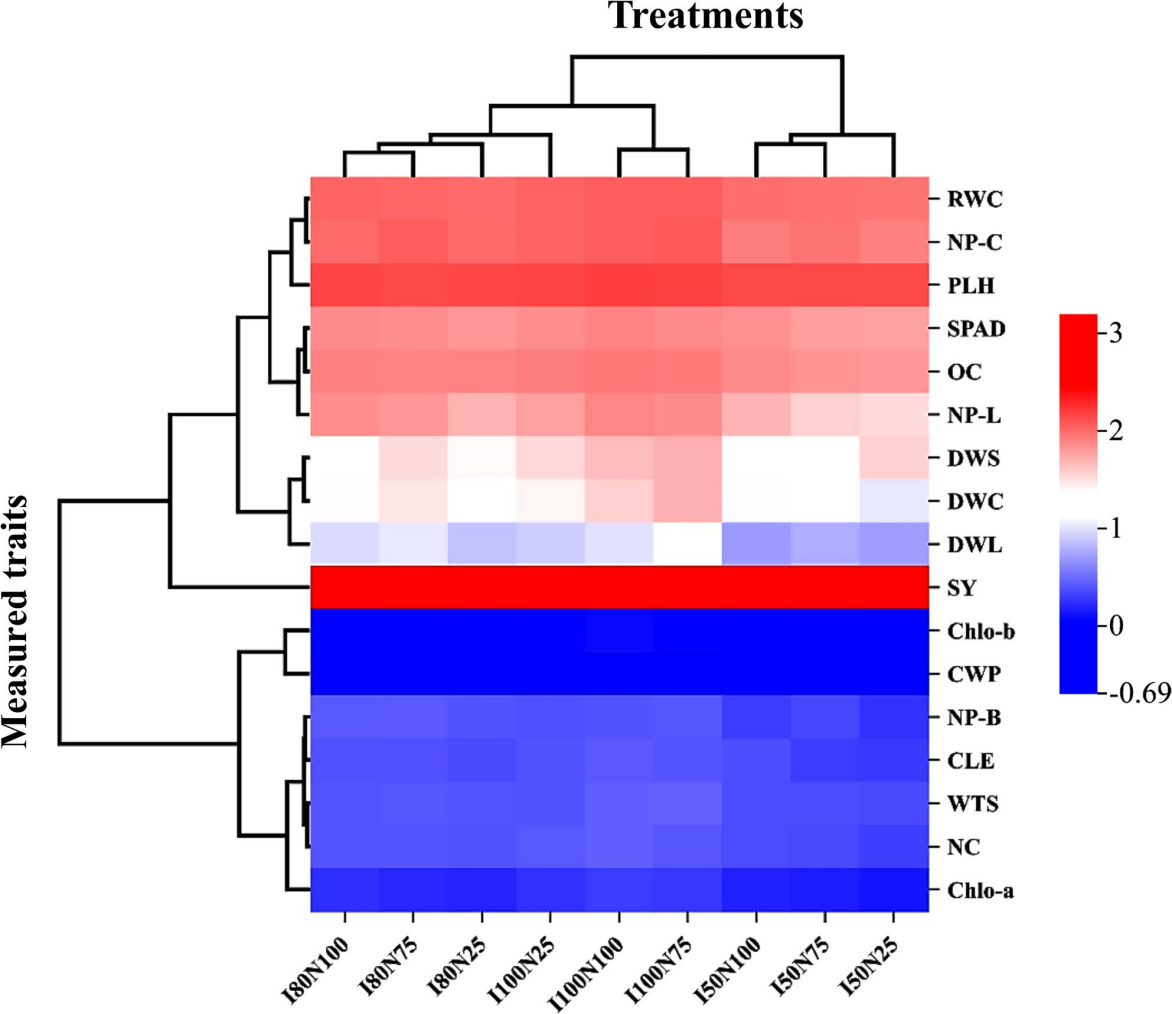
Hierarchical clustering heatmap showing the combined effects of deficit drip irrigation regimes and nitrogen fertigation rates for morpho-physiological, agronomic, and quality properties of sesame under different deficit drip irrigation regimes and nitrogen fertigation rates. PHL = plant height, NP-B = branch number, NP-L = leaf number, DWS = dry weight of stem, DWL = dry weight of leaves, DWC = dry weight of capsule, Chlo-a = chlorophyll a, Chlo-b = chlorophyll b, RWC = relative water content, NP-C = capsule number, CLE = capsule length, WTS = weight of 1000-seed, SY = seed yield, NC = nitrogen content, OC = oil content. CWP = crop water productivity

The interrelationships among the combined treatments (deficit drip irrigation regimes and nitrogen fertigation levels) and the dataset of sesame (including growth, physiological, yield, and quality parameters) were visualized using a clustering heatmap (Fig. 10). The heatmap showed distinct clustering, reflecting differential response patterns between the measured traits. Plant height, capsule number, and relative water content formed a single response cluster, with maximum intensity observed under the well-watered conditions (I_100_) combined with moderate to full nitrogen fertigation rates (N_75_ and N_100_). Furthermore, a coherent response cluster comprising dry weight of stems and capsules exhibited peak intensity at the combination of full irrigation (I_100_) and N_75_ level. This visualization demonstrates that the synergistic effect of adequate water supply and moderately reduced nitrogen input may have optimized resource allocation to reproductive and structural biomass, enhancing stem and capsule development without excess vegetative growth. Remarkably, despite the clear separation into various clusters on the heatmap, physiological parameters (chlorophyll a, chlorophyll b, and relative water content), growth traits (leaf dry weight and leaf number), and nutritional quality attributes (oil and nitrogen content) all exhibited their minimal values under water deficit treatment (I_50_) combined with varying rates of nitrogen fertigation, demonstrating that water availability was the primary limiting factor This suggested that, under water deficit conditions, nitrogen availability, regardless of application rate, was insufficient to mitigate the adverse effects of drought stress on photosynthetic pigment synthesis, vegetative growth, and metabolite accumulation. Consequently, nitrogen use efficiency was likely impaired, and the expected synergistic benefit of nitrogen fertigation was suppressed by the prevailing water limitation, highlighting that water availability acted as the primary limiting factor for these physiological, growth, and quality traits. Moreover, chlorophyll a, branch number, capsule length, 1000-seed weight, and nitrogen content formed a distinct sub-cluster, characterized by their lowest values under stress conditions (I_50_N_25_). This was possibly attributable to the integrated downregulation of photosynthetic capacity, meristematic activity, and reproductive partitioning under the synergistic inhibitory effects of combined water deficit and a low nitrogen fertigation rate. Specifically, limited nitrogen availability may have directly restricted the synthesis of chlorophyll a and nitrogenous biomolecules, while water stress impaired nutrient uptake, transpiration-driven nitrogen transport, and cell expansion. This likely reduced cytokinin biosynthesis, thereby suppressing lateral branching, and limited assimilate allocation to developing capsules, leading to smaller capsules and lighter seeds. Consequently, these traits covary as a coordinated stress-response pattern, reflecting a shift from growth and reproduction toward osmotic adjustment and survival, which collectively explains their lowest values in the heatmap under stress conditions. Overall, hierarchical analysis indicated that I_100_N_75_ was the most balanced treatment, exhibiting optimal performance across most assessed growth- and yield-related attributes in sesame.

## Conclusion

This study investigated the effects of different nitrogen fertigation rates on the morphological, physiological, agronomic, and quality traits of sesame under deficit drip irrigation regimes. The results showed that the I_100_ treatment (full irrigation) maximized plant height and SPAD value, whereas the highest branch number and capsule dry weight were recorded under the N_75_ rate. The integrated application of I_100_ and N_100_ substantially improved photosynthetic pigments (chlorophyll a and b) relative to other treatments. Furthermore, this same treatment combination resulted in the highest relative water content, which was statistically similar to that observed under the interaction between I_100_ and N_100_. Most importantly, full irrigation combined with N_75_ produced the maximum branch number per plant, dry weight and number of capsules per plant, 1000-seed weight, contributing to highest seed yield. In contrast, the stressed conditions (I_50_ and N_25_) minimized the quality attributes (nitrogen and oil content) of sesame seeds, while the highest crop water productivity was documented under synergistic application of I_50_ and N_75_. Future studies should validate these results through multi-location trials using diverse genotypes across various agro-ecological regions to establish standardized irrigation and fertigation recommendations for sesame production. In conclusion, the application of a moderate nitrogen rate (N_75_) under a full irrigation regime is recommended for drip-fertigated sesame to maximize crop productivity and quality.

## Data Availability

All data are included in this article, and any further information will be made available from the corresponding author on reasonable request.

## References

[1] Anastasi U, Sortino O, Tuttobene R, Gresta F, Giuffre A. Agronomic performance and grain quality of sesame (*Sesamum indicum* L.) landraces and improved varieties grown in a Mediterranean environment. Genet Resour Crop Evol. 2017;64:127–37. 10.1007/s10722-015-0338-z.

[2] Ghareeb E, Ismaeil F, Nabarawy A, Eid R. Enhancing growth and yield of sesame plant (*Sesamum indicum* L.) by the amendment of benzyladenine, yeast extract and mixed amino acids. Ann Agric Sci Moshtohor. 2024;62:1–20. 10.21608/assjm.2024.308435.1305.

[3] IMARC Group. Sesame seeds market report by type, color, category, application, distribution channel, and region 2026–2034. GII Research; 2026. https://www.giiresearch.com/report/imarc2032610-sesame-seeds-market-report-by-type-color-category.html. Product Code: 2032610.

[4] FAOSTAT, FAO. Crop production statistics FAO. 2026;:https://www.fao.org/faostat/en/#home

[5] Mekonnen S, Sintayehu A. Performance evaluation of sesame under regulated deficit irrigation application in the low land of Western Gondar, Ethiopia. Int J Agron. 2020;2020:1–9. 10.1155/2020/3760349.

[6] Khasheisiuki A, Shahidi A, Dastorani M, Fallahi H, Shirzadi F. Yield and quality of sesame (*Sesamum indicum* L.) improve by water preservative materials under normal and deficit irrigation in Birjand. Agrotechniques Ind Crop. 2023;3:121–32. 10.22126/ATIC.2023.9167.1098.

[7] Hamoda A, Dabbour M, Lamlom S, El-akshar E. Effectiveness of *Streptomyces enissocaesilis* and chitosan on agronomic, biochemical, and quality traits of soybean under different irrigation intervals. BMC Plant Biol. 2026;26:384. 10.1186/s12870-026-08153-1. 41673555 10.1186/s12870-026-08153-1 PMC12931010

[8] Mareri L, Parrotta L, Cai G. Environmental stress and plants. Int J Mol Sci. 2022;23:5416. 10.3390/ijms23105416. 35628224 10.3390/ijms23105416 PMC9141089

[9] Kaur H, Kohli S, Khanna K, Bhardwaj R. Scrutinizing the impact of water deficit in plants: Transcriptional regulation, signaling, photosynthetic efficacy, and management. Physiol Plant. 2021;172:935–62. 10.1111/ppl.13389. 33686690 10.1111/ppl.13389

[10] Islam F, Gill R, Ali B, Farooq M, Xu L, Najeeb U, et al. Sesame. In: Gupta S, editor. Breeding oilseed crops for sustainable production. Cambridge: Academic; 2016. pp. 135–47. 10.1016/C2013-0-19479-2., Mas sachusetts.

[11] Kouighat M, Hanine H, Fechtali M, Nabloussi A. First report of sesame mutants tolerant to severe drought stress during germination and early seedling growth stages. Plants. 2021;10:1166. 10.3390/plants10061166. 34201345 10.3390/plants10061166 PMC8227276

[12] Hailu E, Urga Y, Sori N, Borona F, Tufa K. Sesame yield response to deficit irrigation and water application techniques in irrigated agriculture, Ethiopia. Int J Agron. 2018;2018:5084056. 10.1155/2018/5084056.

[13] Liu S, Cui S, Adamowski J, Wu N, Wu M, Zhang P, et al. Assessing the impact of various irrigation technologies on agricultural production: A water-energy–carbon nexus perspective. Sci Total Environ. 2024;954:176809. 10.1016/j.scitotenv.2024.176809. 39395485 10.1016/j.scitotenv.2024.176809

[14] Akin F, Cemek B. The effects of different water deficit levels on yield and water use productivity of sesame irrigated by subsurface drip irrigation. Irrig Sci. 2024;43:995–1013. 10.1007/s00271-025-01033-w.

[15] Hamed S, EL-Desouky H, El-Dayem H, Abomarzoka E, Eid R. Effect of foliar application with putrescine and benzyladenine on vegetative growth, yield and its components and seed oil contents of chia (*Salvia hispanica* L.) plants grown under water stress conditions. Ann Agric Sci Moshtohor. 2023;61:2023. 10.21608/ASSJM.2023.326714.

[16] Guo H, Li S. A Review of drip irrigation’s effect on water, carbon fluxes, and crop growth in farmland. Water. 2024;16:2206. 10.3390/w16152206.

[17] Bastug R, Karaca C, Buyuktas D, Aydinsakir K, Dinc N. The effects of deficit irrigation practices on evapotranspiration, yield and quality characteristics of two sesame varieties (*Sesamum indicum* L.) grown in lysimeters under the Mediterranean climate conditions. Irrig Sci. 2021;39:587–606. 10.1007/s00271-021-00732-4.

[18] Yang P, Wu L, Cheng M, Fan J, Li S, Wang H, et al. Review on drip irrigation: Impact on crop yield, quality, and water productivity in China. Water. 2023;15:1733. 10.3390/w15091733.

[19] Couch A, Jani A, Mulvaney M, Hochmuth G, Bennett J, Gloaguen R, et al. Nitrogen accumulation, partitioning, and remobilization by diverse sesame cultivars in the humid southeastern USA. F Crop Res. 2017;203:55–64. 10.1016/j.fcr.2016.12.012.

[20] Yan F, Zhang F, Fan X, Fan J, Wang Y, Zou H, et al. Determining irrigation amount and fertilization rate to simultaneously optimize grain yield, grain nitrogen accumulation and economic benefit of drip-fertigated spring maize in northwest China. Agric Water Manag. 2021;243:106440. 10.1016/j.agwat.2020.106440.

[21] Alipour Babadi M, Masir M, Moezzi A, Rahnama A, Taghavi M. Iron (Fe) biofortification using Fe-Aminochelates in sunflower growing in calcareous soils. J plant Nutr soil Sci. 2025;188:519–35. 10.1002/jpln.12004.

[22] Song J, Yang H, Yu X, Chen A, Yang C, He Y, et al. Effects of combined application of nitrogen, phosphorus, and potassium fertilizers on seed yield, seed quality and economic returns of Elymus nutans in alpine region. BMC Plant Biol. 2025;25:130. 10.1186/s12870-025-06126-4. 39885376 10.1186/s12870-025-06126-4 PMC11781048

[23] Zenawi G, Mizan A. Effect of nitrogen fertilization on the growth and seed yield of sesame (*Sesamum indicum* L). Int J Agron. 2019;5027254. 10.1155/2019/5027254.

[24] Blal A, Kamel S, Mahfouz H, Abd El-Wahed M. Impact of pollination and fertilization on sesame production in the reclaimed lands, Ismailia governorate, Egypt. J Agric Sci. 2012;57:121–33. 10.2298/JAS1203121B.

[25] Jackson ML. Soil chemical analysis. Prentice Hall of India. New Delhi: Pvt. Ltd.; 1967.

[26] Klute A, Dirksen C. Hydraulic conductivity and diffusivity: laboratory methods. In: in Methods of soil analysis, Madison, USA: American Society of Agronomy, Crop Science Society of America, and Soil Science Society of America; 1986. pp. 687–734. 10.2136/sssabookser5.1.2ed.c28

[27] Page A, Miller R, Keeney D. Methods of soil analysis. Chemical and microbiological properties. Madison, USA: Soil Science Society of America; 1982. 10.2134/agronmonogr9.2.2ed.

[28] Gardner W. Water content. In: Klute A, editor. Methods of soil analysis: Part 1 Physical and mineralogical methods. American Society of Agronomy, Inc. Soil Science Society of America, Inc.; 1986. pp. 493–544. 10.2136/sssabookser5.1.2ed.c21.

[29] Mourad K, Ismail Y, Othman M, Kandeel D, Abdelghany M. Assessing the drought tolerance of some sesame genotypes using agro-morphological, physiological, and drought tolerance indices. BMC Plant Biol. 2025;25:352. 10.1186/s12870-025-06235-0. 40098085 10.1186/s12870-025-06235-0 PMC11917027

[30] Vermeiren L, Jobling G. Localized irrigation: Design, installation, operation, evaluation. Rome: FAO; 1980.

[31] Mohamoud A, Abdalla S, Elhag M, Yousif L. Estimation of water requirement and water productivity of sesame crop (*Sesamum indicum* L.) in dryland areas of Sennar State, Sudan. Sudan J Des Res. 2019;11:1–16.

[32] Arnon DI. Copper enzymes in isolated chloroplasts. Polyphenoloxidase in *Beta Vulgaris*. Plant Physiol. 1949;24:1–15. 10.1104/pp.24.1.1. 16654194 10.1104/pp.24.1.1 PMC437905

[33] González L, González-Vilar M. Determination of relative water content. In: Reigosa Roger MJ, editor. Handbook of plant ecophysiology techniques. Dordrecht: Springer; 2001. pp. 207–12. 10.1007/0-306-48057-3_14.

[34] Hamoda A, Dabbour M. Optimizing corn productivity: Hybrid and intra-row spacing effects on growth, yield, and nutritional quality. Sci Rep. 2025;15:32601. 10.1038/s41598-025-19439-z. 40968154 10.1038/s41598-025-19439-z PMC12446486

[35] Hamoda A, Khojah E, Radhi K. Synergistic effects of herbicides and gibberellic acid on wheat yield and quality. Sci Rep. 2025;15:7496. 10.1038/s41598-025-90217-7. 40032875 10.1038/s41598-025-90217-7 PMC11876365

[36] Michael A. Irrigation theory and practice. New Delhi: Vi_kas Publishing House; 1978.

[37] Ajithkumar I, Panneerselvam R. Osmolyte accumulation, photosynthetic pigment and growth of *Setaria italica* (L.) P. Beauv. under drought stress. Asian Pac J Reprod. 2013;2:220–4. 10.1016/S2305-0500(13)60151-7.

[38] Malam K, Solanki R. Growth, yield and water use efficiency of sweet sorghum [*Sorghum bicolor* (L.) Moench] affected by drip irrigation and nitrogen levels through fertigation. Int J Environ Clim Chang. 2022;12:2407–24. 10.9734/IJECC/2022/v12i1131234.

[39] Jadhav S, Naiknaware M, Pawar G. Effect of nitrogen, phosphorus and biofertilizer on growth, yield and quality of summer sesame. Int J Trop Agric Ser Publ. 2015;33:475–80.

[40] Lu X, Dijkstra F, Kong D, Wang Z, Han X. Plant nitrogen uptake drives responses of productivity to nitrogen and water addition in a grassland. Sci Rep. 2014;4:4817. 10.1038/srep04817. 24769508 10.1038/srep04817 PMC4001094

[41] Kuscu H, Turhan A, Ozmen N, Aydinol P, Demir A. Optimizing levels of water and nitrogen applied through drip irrigation for yield, quality, and water productivity of processing tomato (*Lycopersicon esculentum* Mill). Hort Environ Biotechnol. 2014;55:103–14. 10.1007/s13580-014-0180-9.

[42] Zhao W, Liu L, Shen Q, Yang J, Han X, Tian F, et al. Effects of water stress on photosynthesis, yield, and water use efficiency in winter wheat. Water. 2020;12:2127. 10.3390/w12082127.

[43] El-Mageed A, Ibrahim E, Mahmoud S. Use of botanical traits and molecular markers for assessment of induced variations via gamma rays in M2 and M3 sesame mutated generations. Egypt J Plant Breed. 2016;20:1119–34.

[44] Tabassum D, Akhtar A, Inam A. Effect of waste water irrigation on growth,physiology,and yield of mustard. Int J Bot Res. 2013;3:27–34.

[45] Qiao M, Hong C, Jiao Y, Hou S, Gao H. Impacts of drought on photosynthesis in major food crops and the related mechanisms of plant responses to drought. Plants. 2024;13:1808. 10.3390/plants13131808. 38999648 10.3390/plants13131808 PMC11243883

[46] Liu H, Wang X, Wang D, Zou Z, Liang Z. Effect of drought stress on growth and accumulation of active constituents in *Salvia miltiorrhiza* Bunge. Ind Crop Prod. 2011;33:84–8. 10.1016/j.indcrop.2010.09.006.

[47] Wichaidist B, Intrman A, Junhom P, Puttrawutichai S. Environmental and sustainability indicators the effect of irrigation practices on stress-related physiological characteristics, water productivity, and water footprint in rice cultivation. Environ Sustain Indic. 2025;26:100695. 10.1016/j.indic.2025.100695.

[48] Jan A, Hadi F, Ditta A, Suleman M, Ullah M. Zinc-induced anti-oxidative defense and osmotic adjustments to enhance drought stress tolerance in sunflower (*Helianthus annuus* L). Environ Exp Bot. 2022;193:104682. 10.1016/j.envexpbot.2021.104682.

[49] Kishor PB, Hong Z, Miao C, Hu C, Verma D. Overexpression of ∆1-Pyrroline-5-Carboxylate synthetase increases proline production and confers osmotolerance in transgenic plants. Plant Physiol. 1995;108:1387–94. 10.1104/pp.108.4.1387. 12228549 10.1104/pp.108.4.1387 PMC157516

[50] Sravanthi A, Ratnakumar P, Narender S, Reddy S, Eswari K, Pandey B, et al. Morpho-physiological, quality traits and their association with seed yield in sesame (*Sesamum indicum* L.) indigenous collection under deficit moisture stress. Plant Physiol Rep. 2022;27:132–42. 10.1007/s40502-021-00621-0.

[51] Rahnama A, James R, Poustini K, Munns R. Stomatal conductance as a screen for osmotic stress tolerance in durum wheat growing in saline soil. Funct Plant Biol. 2010;37:255–63. 10.1071/FP09148.

[52] Tombesi S, Nardini A, Frioni T, Soccolini M, Zadra C, Farinelli D, et al. Stomatal closure is induced by hydraulic signals and maintained by ABA in drought-stressed grapevine. Sci Rep. 2015;5:12449. 10.1038/srep12449. 26207993 10.1038/srep12449 PMC4513549

[53] Patmi YS, Pitoyo A, Solichatun S. Effect of drought stress on morphological, anatomical, and physiological characteristics of Cempo Ireng Cultivar Mutant Rice (*Oryza sativa* l.) strain 51 irradiated by gamma-ray. J Phys Conf Ser IOP Publ. 2020;1436(1):012015. 10.1088/1742-6596/1436/1/012015.

[54] Chauhan S, Rao V, Reddy A, Jayasree G, Reddy S. Response of sesame (*Sesamum indicum* L.) to irrigation scheduling based on climatological approach and N fertigation levels. J Oilseeds Res. 2016;33:38–44. 10.56739/jor.v33i1.139039.

[55] Ali A, Jabeen N, Farruhbek R, Chachar Z, Laghari A, Chachar S, et al. Enhancing nitrogen use efficiency in agriculture by integrating agronomic practices and genetic advances. Front Plant Sci. 2025;16:1–13. 10.3389/fpls.2025.1543714. 10.3389/fpls.2025.1543714 PMC11951869 40161228

[56] Ucan K, Kıllı F, Gencoglan C, Merdun H. Effect of irrigation frequency and amount on water use efficiency and yield of sesame (*Sesamum indicum* L.) under field conditions. F Crop Res. 2007;101:249–58. 10.1016/j.fcr.2006.11.011.

[57] Nadeem A, Kashani S, Ahmed N, Buriro M, Saeed Z, Mohammad F, et al. Growth and yield of sesame (*Sesamum indicum* L.) under the influence of planting geometry and irrigation regimes. Am J Plant Sci. 2015;6:980–6. 10.4236/ajps.2015.67104.

[58] El-Tantawy M, Ouda A, Khalil A. Irrigation scheduling for maize grown under Middle Egypt conditions. Res J Agric Biol Sci. 2007;3:456–62.

[59] Jeyaraj S, Raj K, Puthur J, Beevy S. Differential physio-chemical responses of wild and cultivar sesamum species exposed to drought and recovery. South Afr J Bot. 2024;172:430–47. 10.1016/j.sajb.2024.07.031.

[60] Naim A, Ahmed M. Effect of irrigation on vegetative growth, oil yield and protein content of two sesame (*Sesamum indicum* L.) cultivars. Res J Agric Biol Sci. 2010;6:630–6.

[61] Kadkhodaie A, Razmjoo J, Zahedi M, Pessarakli M. Oil content and composition of sesame (*Sesamum indicum* L.) genotypes as affected by irrigation regimes. J Am Oil Chem Soc. 2014;91:1737–44. 10.1007/s11746-014-2524-0.

[62] Fazeli F, Ghorbanli M, Niknam V. Effect of drought on biomass, protein content, lipid peroxidation and antioxidant enzymes in two sesame cultivars. Biol Plant. 2007;51:98–103. 10.1007/s10535-007-0020-1.

[63] Thanki R, Solanki R, Modhavadia J, Gohil B, Prajapati P. Effect of irrigation scheduling at critical growth stages and fertility levels on growth, yield and quality of summer sesame (*Sesamum indicum* L). Indian Soc Oilseeds Res. 2014;31:41–5.

[64] Alipourbabadi M, Norouzimasir M, Moezzi A, Rahnama A, Taghavi M, Rashtbari M, et al. Aminochelates drive the reorganization of root-associated enzyme hotspots in the sunflower rhizosphere. Rhizosphere. 2026;37:101299. 10.1016/j.rhisph.2026.101299.

[65] Tas I. Evaluation of irrigation experiments with GGE biplot method and economic analysis of drip irrigation system: A case study of peanut production. 2023;29:464–77. 10.15832/ankutbd.1124344

[66] Desoky E, Alharbi K, Rady M, Elnahal A, Selem E, Arnaout S, et al. Physiological, biochemical, anatomical, and agronomic responses of sesame to exogenously applied polyamines under different irrigation regimes. Agronomy. 2023;13:875. 10.3390/agronomy13030875.

[67] Tura L, Tolossa T. Systematic review: Effect of irrigation water quality and deficit irrigation on crop yield and water use efficiency. Turkish J Agric - Food Sci Technol. 2020;8:1201–10. 10.24925/turjaf.v8i5.1201-1210.3366.

[68] Hamoda A, El-mehy A, Dabbour M, Arab Z. Effect of faba bean-garlic intercropping on low-molecular-weight organic acids, yield components, and profitability under different spatial arrangements. Sci Rep. 2026;16:13888. 10.1038/s41598-026-49974-2. 42062419 10.1038/s41598-026-49974-2 PMC13133401

[69] Dabbour M, Fikry M, Lamlom S, Wu P, He R, Rashid A, et al. Modifying cottonseed protein: Effect of sonication and localized proteolysis on molecular conformation, thermal stability, and solubility. Food Hydrocoll. 2026;180:112911. 10.1016/j.foodhyd.2026.112911.

[70] Dabbour M, Hamoda A, Xu H, Mintah BK, Wahia H, Betchem G, et al. pH-shifting and sonication synergistically altered cottonseed protein: Correlating the conformational and functional characteristics. Ind Crops Prod. 2024;222:120043. 10.1016/j.indcrop.2024.120043.

[71] Dabbour M, Hamoda A, Wu P, He R, Wahia H, Betchem G, et al. Functionalizing cottonseed protein by sequential pH-shifting and ultrasonication. Int J Biol Macromol. 2025;323:147207. 10.1016/j.ijbiomac.2025.147207. 40885355 10.1016/j.ijbiomac.2025.147207

[72] Hamoda A. Effect of nano-fertilizer and bio-growth regulator on yield attributes of wheat. J Plant Prod. 2024;15:101–9. 10.21608/jpp.2024.266830.1305.

